# Differential gene expression in liver and small intestine from lactating rats compared to age-matched virgin controls detects increased mRNA of cholesterol biosynthetic genes

**DOI:** 10.1186/1471-2164-12-95

**Published:** 2011-02-03

**Authors:** Antony Athippozhy, Liping Huang, Clavia Ruth Wooton-Kee, Tianyong Zhao, Paiboon Jungsuwadee, Arnold J Stromberg, Mary Vore

**Affiliations:** 1 Graduate Center for Toxicology, University of Kentucky, Lexington, Kentucky, 40536 USA; 2 Department of Statistics, University of Kentucky, Lexington, Kentucky, 40506; USA

## Abstract

**Background:**

Lactation increases energy demands four- to five-fold, leading to a two- to three-fold increase in food consumption, requiring a proportional adjustment in the ability of the lactating dam to absorb nutrients and to synthesize critical biomolecules, such as cholesterol, to meet the dietary needs of both the offspring and the dam. The size and hydrophobicity of the bile acid pool increases during lactation, implying an increased absorption and disposition of lipids, sterols, nutrients, and xenobiotics. In order to investigate changes at the transcriptomics level, we utilized an exon array and calculated expression levels to investigate changes in gene expression in the liver, duodenum, jejunum, and ileum of lactating dams when compared against age-matched virgin controls.

**Results:**

A two-way mixed models ANOVA was applied to detect differentially expressed genes. Significance calls were defined as a p < 0.05 for the overall physiologic state effect (lactation vs. control), and a within tissue pairwise comparison of p < 0.01. The proportion of false positives, an estimate of the ratio of false positives in the list of differentially expressed genes, was calculated for each tissue. The number of differentially expressed genes was 420 in the liver, 337 in the duodenum, 402 in the jejunum, and 523 in the ileum. The list of differentially expressed genes was in turn analyzed by Ingenuity Pathways Analysis (IPA) to detect biological pathways that were overrepresented. In all tissues, sterol regulatory element binding protein (Srebp)-regulated genes involved in cholesterol synthesis showed increased mRNA expression, with the fewest changes detected in the jejunum. We detected increased Scap mRNA in the liver only, suggesting an explanation for the difference in response to lactation between the liver and small intestine. Expression of *Cyp7a1*, which catalyzes the rate limiting step in the bile acid biosynthetic pathway, was also significantly increased in liver. In addition, decreased levels of mRNA associated with T-cell signaling were found in the jejunum and ileum. Several members of the Solute Carrier (SLC) and Adenosine Triphosphate Binding Cassette (ABC) superfamilies of membrane transporters were found to be differentially expressed; these genes may play a role in differences in nutrient and xenobiotic absorption and disposition. mRNA expression of *SLC39a4_predicted*, a zinc transporter, was increased in all tissues, suggesting that it is involved in increased zinc uptake during lactation. Microarray data are available through GEO under GSE19175.

**Conclusions:**

We detected differential expression of mRNA from several pathways in lactating dams, including upregulation of the cholesterol biosynthetic pathway in liver and intestine, consistent with Srebp activation. Differential T-Cell signaling in the two most distal regions of the small intestine (ileum and jejunum) was also noted, as well as differential expression of transporters that likely play a key role in nutrient uptake.

## Background

Lactation is a time of a four- to five-fold increased energy demand imposed by the suckling young that requires a proportional adjustment in the ability of the lactating dam to absorb nutrients and to synthesize critical biomolecules to meet the dietary needs of both the offspring and the dam [1-3]. Lactating rats have a two- to three-fold increase in food consumption (hyperphagia) [1-3], in part through the decreased suppression of appetite accompanying decreased serum leptin [4].

Diet and hyperphagia have been shown to influence the rate of cholesterol synthesis, which is increased in the liver and small intestine in the lactating rat [5,6]. Of these tissues, the liver is the primary contributor to serum levels of cholesterol, and shows a quantitatively greater increase in the rate of cholesterol synthesis during lactation [5]. 3-Hydroxy-3-methylglutaryl-coenzyme A reductase (Hmgcr), the enzyme catalyzing the rate-limiting step of cholesterol synthesis, shows significantly increased activity in the liver during lactation compared to virgin and nonlactating control rats [7,8]. Cholesterol synthetic and lipogenic genes are regulated by transcription factors termed sterol regulatory element binding proteins (Srebp); the activity of Srebp proteins is in turn regulated by the Srebf chaperone (Scap) and the Insulin induced genes (Insig).

Circulating serum levels of several hormones that regulate metabolism are decreased during lactation in the rat, including thyroid hormone, insulin and leptin [4]. Such changes in hormone signaling and diet are likely to have large influences on the activation of their corresponding pathways. Receptors for leptin, thyroid hormone, and insulin are expressed in both the liver and small intestine [9-14], with liver being considered a major site of insulin signaling [9] and thyroid receptor β (TRB, Thrb) being the dominant form of the thyroid receptor in both tissues [14]. Leptin acts on the small intestine and inhibits sugar uptake [11], and the liver is a major source of the soluble form of the leptin receptor, particularly under conditions of negative energy balance [12], as occurs in lactation [1]. Therefore, altered serum levels of these hormones would be expected to influence mRNA expression of downstream genes.

Our laboratory has been investigating the effects of lactation on the synthesis and transport of bile acids in the liver and small intestine in the rat [15]. Bile acids are synthesized in the liver from cholesterol, and are essential for the biliary excretion of cholesterol and for the efficient intestinal absorption of cholesterol, lipid-soluble vitamins and lipids [16]. Bile acids are secreted into bile by the Bile salt export pump (Bsep; Abcb11), taken up across the apical membrane of the enterocyte in the terminal ileum by the Apical sodium-dependent bile acid transporter (Asbt; Slc10a2), effluxed into portal blood by the Organic solute transporter heterodimer (Ostα/β), and then taken up in the hepatocyte by the Sodium-dependent taurocholate co-transporting polypeptide (Ntcp; Slc10a1) [17]. Expression of Ntcp, Bsep and Asbt are all increased in lactation [18,19], as is the size of the bile acid pool [15]. We recently demonstrated that expression and activity of Cyp7a1, the enzyme catalyzing the rate limiting step in the conversion of cholesterol to bile acids, is increased at mid-lactation (day 10 - 14 postpartum) [15]. Further, this increase occurs at 16 h (10 h of light on a 12 h/light dark cycle; 4 PM) and represents a shift in the diurnal rhythm of *Cyp7a1 *expression, which is normally maximal in the dark cycle (i.e., 22 h). Increased expression of *Cyp7a1 *is apparently due to decreased expression of Fibroblast Growth Factor 15 (*Fgf15*) in the ileum, resulting in decreased Fgf15 signaling via Fibroblast Growth Factor Receptor 4 (Fgfr4) and Erk1/2 in liver and decreased repression of *Cyp7a1 *transcription [20].

In order to identify further changes in expression of genes important in the regulation of bile acid and cholesterol synthesis, as well as other genes important in meeting nutritional demands and physiological changes of the lactating rat, we carried out a microarray experiment in the liver and small intestine of the lactating dam at 16 h on days 10 -11 postpartum and compared these to gene expression in female virgin control rats.

## Results

### Detection of differentially expressed genes

A repeated measures mixed model ANOVA was used to test for effects of tissue and lactation, as described in Methods. A statistically significant difference was determined to exist when the physiologic state effect (comparison between all control samples against all lactating samples) yielded a p < 0.05 and the effect of lactation within a given tissue (physiologic state simple effect) yielded a p < 0.01. Analyzed data are available in Additional File 1: Statistical_Analysis_and_Statistical_Pattern_Matching_Results.txt. These p-values were used as cutoffs for differentially expressed genes and led to the proportions of false positives that are listed in the section titled "Approximation of false discoveries" below.

A number (1,114) of genes demonstrated an interaction at p < 0.01 and 556 genes passed a Benjamini-Hochberg false discovery rate correction at FDR = 0.05 (Additional File 2: Benjamini_Hochberg_False_Discovery_Rates.txt). These genes represent those that displayed a different response to lactation in one tissue with respect to the other tissues. However, it should be noted that this list was not a useful cutoff, as genes that responded uniformly to lactation across all tissues would be ignored. Also, five genes that passed the Benjamini-Hochberg correction did not show any significant changes at p < 0.01 when the effect of lactation was tested within each tissue. Since the primary purpose of this study was to characterize the influence of lactation on gene expression on these tissues, the interaction term was not used. The tissue effect p-values indicated that many (70% at p < 0.01) genes were differentially expressed across tissues, due to the large difference in cell types between the liver and the small intestine. Therefore, we chose the cutoffs of a physiologic state main effect at p < 0.05 and the effect of lactation within at least one tissue at p < 0.01, as described in the Methods.

Although not as many genes were detected as significantly differentially expressed compared to the overall tissue effect, the overall physiologic state effect and the pairwise comparisons (effect of lactation in each tissue) showed a high number of low p-values, indicating that the tissues in question responded to lactation at the level of mRNA. Several genes were downregulated in the duodenum only (34 genes), with 23 genes showing over a 50% decrease. Members of this group are listed as pattern "-100" in Additional File 1. Histograms displaying the distribution of p-values are in Additional File 3: Histograms_of_p_values.ppt , and volcano plots displaying each tissue's response to lactation are in Additional File 4: Volcano_plots.doc.

### Approximation of false discoveries

The proportion of false positives is an approximation of the ratio of false positives in the list of genes listed as differentially expressed. An estimate of the number of false positives was calculated for all tissues using genes where p < 0.05 for an overall physiologic state effect, and for each tissue using genes with an effect of lactation within each tissue (here defined as a simple effect) of p < 0.01. Thus, the proportion of false positives (PFP) was calculated as (p-value cutoff × number of genes tested)/number of genes detected below the p-value cutoff. The PFP as defined by Fernando et al [21], is E(V)/E(R) where E(V) is the expected number of false rejections of the null hypothesis and E(R) is the expected number of rejections of the null hypothesis. Here we utilized the actual number of rejections of the null hypothesis as the expected value. Of all of the genes on the chip, 14,129 genes were found to be annotated and expressed in at least one tissue/physiologic-state combination and were used for statistical tests. Of those genes, 1,924 had an overall physiologic state p-value of less than 0.05, yielding a PFP of 0.37; at an overall physiologic state p < 0.01, 690 genes were detected, yielding a PFP of 0.20. PFPs for the individual tissues at a cutoff of p < 0.01 for the pairwise comparisons (simple effects) were 0.17 for the liver, 0.28 for the duodenum, 0.22 for the jejunum, and 0.18 for the ileum. The PFPs for the individual tissue calculations examined only the genes detected at a simple effect p < 0.01, and not at the combined overall physiologic state cutoff of p < 0.05 together with the within-tissue cutoff of p < 0.01, as some genes passed the p < 0.01 cutoff within a given tissue, but did not pass the initial overall physiologic state effect cutoff of p < 0.05. The rationale for not utilizing the overall physiologic state effect together with the within tissue physiologic state effect in the calculation was that these multiple tests were utilized for the same gene. Consequently, the list of genes reported at both p < 0.05 for the physiologic state and p < 0.01 for the comparison within a tissue was a subset of the list of genes that only show a p < 0.01 within a tissue. The physiologic state cutoff of p < 0.05 was chosen to protect against repeated testing for each tissue; this value was also chosen because changes that only occurred within one tissue would be difficult to detect if the overall physiologic state cutoff was made at p < 0.01. Approximations of the proportion of false positives in this range (0.17 - 0.28) have been reported previously [22].

### RT-PCR Validation of Microarray Data

Results from RT-PCR analyses agreed with the trends detected in the microarray analyses (Table 1). In some cases, significance calls differed, but the directionality of the changes observed was consistent with the microarray data. Possible causes for disagreement included the fact that different methods of normalization were used between RT-PCR and the microarray.

**Table 1:** RT-PCR validation of selected genes from the microarray

RT-PCR Measurements	Transcript Cluster ID	RL	RD	RJ	RIL
Tmem97	7080099	1.52 (p = 0.021)	2.60 (p < 0.01)	1.59 (p = 0.42)	1.73 (p = 0.072)

Npc1l1	-	1.00 (p = 1.00)	1.27 (p = 0.49)	1.22 (p = 0.16)	0.88 (p = 0.72)

Abcb1a	7250393	0.82 (p = 0.91)	0.83 (p = 0.77)	0.72 (p = 0.09)	0.65 (p < 0.01)

Cyp1a1	7336681	2.98 (p = 0.93)	1.12 (p = 0.64)	2.06 (p < 0.01)	0.78 (p = 0.89)

Cyp3a23/3a1	7100149	1.83 (p = 0.12)	1.30 (p = 1.00)	BDL	BDL

Fdft1	7139070	1.36 (p = 0.11)	2.58 (p = 0.22)	1.02 (p = 0.99)	2.41 (p = 0.018)

Hmgcr	7202670	2.05 (p < 0.01)	1.52 (p = 0.30)	1.14 (p = 0.80)	1.18 (p = 0.48)

Slc39a4	7329323	2.71 (p = 0.59)	2.92 (p < 0.01)	2.20 (p < 0.01)	1.99 (p < 0.01)

Sqle	7317317	1.80 (p < 0.01)	2.66 (p = 0.034)	1.71 (p = 0.50)	1.59 (p = 0.056)

Ugt2b36 (Ugt2b4)	7117373	0.85 (p = 0.047)	0.76 (=0.41)	0.39 (p = 0.21)	0.62 (p = 1.00)

**Microarray** **Mean Log2** **Measurements**		**RL**	**RD**	**RJ**	**RIL**

Tmem97	7080099	1.92 (p < 0.01)	2.03 (p < 0.01)	1.82 (p < 0.01)	2.15 (p < 0.01)

Abcb1a	7250393	0.63 (p < 0.01)	0.66 (p < 0.01)	0.65 (p < 0.01)	0.70 (p = 0.01)

Cyp1a1	7336681	2.29 (p < 0.01)	1.23 (p = 0.35)	1.71 (p = 0.02)	0.67 (p = 0.03)

Cyp3a23/3a1	7100149	1.69 (p < 0.01)	0.98 (p = 0.81)	1.05 (p = 0.50)	1.04 (p = 0.58)

Fdft1	7139070	1.38 (p < 0.01)	1.98 (p < 0.01)	1.42 (p < 0.01)	1.55 (p < 0.01)

Hmgcr	7202670	1.97 (p < 0.01)	1.64 (p < 0.01)	1.17 (p = 0.14)	1.38 (p < 0.01)

Slc39a4	7329323	2.81 (p < 0.01)	1.89 (p < 0.01)	1.65 (p < 0.01)	1.68 (p < 0.01)

Sqle	7317317	1.83 (p < 0.01)	2.27 (p < 0.01)	1.72 (p < 0.01)	1.70 (p < 0.01)

Ugt2b36 (Ugt2b4)	7117373	0.96 (p = 0.86)	0.57 (p < 0.01)	0.37 (p < 0.01)	1.00 (p = 0.97)

Ratios were calculated as Lactating measurement divided by Control measurement. RT-PCR data is reported as the ratio of normalized measurements, and microarray data is reported as the ratio of normalized, untransformed intensities. p-Values were calculated using a mixed models approach as described in methods. RL, RD, RJ and RIL are the Ratio of Lactation to Control in liver (L), duodenum (D), jejunum (J), and ileum (IL), respectively. BDL, below detection limit

### Patterns

Patterns were identified using statistical pattern matching [23,24] by assigning each gene as significantly "up", "down", or "no change" detected in each tissue. The results of the statistical pattern matching showed that fifteen genes were upregulated in all four tissues, while thirty-one genes were downregulated in all four tissues. Seventy-two genes were upregulated in liver only, and another ninety-nine genes were downregulated in liver only. Results from analyses of each pattern using DAVID [25] are shown in additional file 5: DAVID_output_file.txt.

Of the fifteen genes upregulated in every tissue (Table 2), seven were identified by DAVID as being involved in the Biosynthesis of Sterols pathway, where p = 4.59 × 10^-13^, using the list of "Up in All Tissues" for the DAVID analysis. One gene, *transmembrane protein 97 *(*Tmem97*), has been identified as being regulated by the Srebp proteins [26,27], and was recently suggested to aid in Low density lipoprotein receptor (Ldlr) function [26]. Another gene, *RNA (guanine 9) methyltransferase domain containing 2 *(*Rg9mtd2*), is the homolog for a tRNA methyl transferase that occurs in yeast [28]. *Slc39a4_predicted *was upregulated in all tissues, consistent with increased zinc absorption during lactation, and is discussed further below.

**Table 2:** Genes that displayed increased mRNA during lactation in all tissues

Transcript Cluster ID	Gene Name	RL	RD	RJ	RIL
7027017	Acetyl-Coenzyme A acetyltransferase	1.98 (p = 0.0173)	2.40 (p = 0.0049)	1.80 (p = 0.0363)	2.19 (p = 0.0128)

7080099	Transmembrane protein 96	1.92 (p = 0.0001)	2.03 (p = 2.8e-05)	1.82 (p = 0.0002)	2.15 (p = 8.7e-06)

7113785	Hydroxysteroid(17-beta) dehydrogenase 7	1.71 (p = 2.1e-06)	1.54 (p = 4.7e-05)	1.19 (p = 0.045)	1.51 (p = 0.0001)

7132836	PDZ binding kinase predicted*	1.56 (p = 0.0222)	1.88 (p = 0.0031)	1.66 (p = 0.0054)	1.54 (p = 0.0227)

7139070	Farnesyl diphosphate farnesyl transferase	1.38 (p = 0.0009)	1.98 (p = 4.7e-08)	1.42 (p = 0.0004)	1.55 (p = 4.1e-05)

7144691	Sterol-C4-methyl oxidase-like	1.41 (p = 0.0015)	2.19 (p = 1.9e-07)	1.45 (p = 0.0008)	1.80 (p = 8.2e-06)

7166170	Isopentenyl-diphosphate delta isomerase	1.61 (p = 0.0006)	2.11 (p = 2.8e-06)	1.33 (p = 0.0226)	1.91 (p = 2.6e-05)

7169182	Kinesin family member 20a_predicted*	1.46 (p = 0.0012)	1.50 (p = 0.0010)	1.33 (p = 0.0104)	1.45 (p = 0.0013)

7186293	Mevalonate (diphospho) decarboxylate	2.65 (p = 3.8e-05)	2.15 (p = 0.0004)	1.49 (p = 0.0316)	1.73 (p = 0.0042)

7199743	RNA (guanine-9-) methyltransferase domain containing 2	1.18 (p = 0.009)	1.42 (p = 3.6e-06)	1.19 (p = 0.0066)	1.30 (p = 0.002)

7250653	CYP51	1.24 (p = 0.0116)	2.14 (p = 1.8e-09)	1.50 (p = 4.2e-05)	1.89 (p = 6.7e-08)

7280610	UDP-Galactose-4-epimerase	1.65 (p = 0.0006)	1.50 (p = 0.0042)	1.36 (p = 0.0243)	1.53 (p = 0.0023)

7291805	Dehydrodolichyl diphosphate synthase	1.37 (p = 0.0003)	1.36 (p = 0.0005)	1.27 (p = 0.0041)	1.17 (p = 0.0447)

7317317	Squalene epoxidase	1.83 (p = 2.4e-05)	2.27 (p = 5.6e-07)	1.72 (p = 0.0001)	1.70 (p = 0002)

7329323	Solute carrier 39 (zinc transporter) member 4	2.81 (p = 2.5e-10)	1.89 (p = 9.9e-08)	1.65 (p = 2.1e-06)	1.68 (p = 1.2e-06)

To be considered part of a grouping, genes must have had a physiologic state p < 0.05 and at least one tissue simple effect p < 0.01. Reported p-values are the tissue simple effect p-values. The tissue simple effect p-value represents the comparison between Lactation and Control in the corresponding tissue. For the purposes of assigning patterns, the significance cutoff for the remaining tissues' simple effect p-values was set to p < 0.05. Abbreviations used as in Table 1. *Gene is at the Extended confidence level.

**Figure 1:**
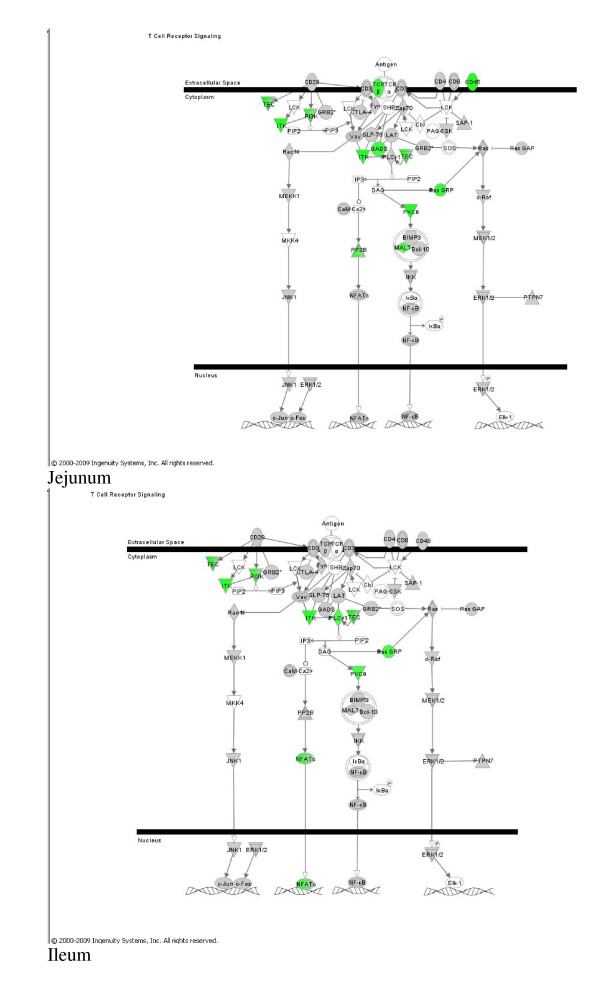
**T-cell signaling in lactating jejunum and ileum**. Downregulation of T-cell signaling in the jejunum (top, p = 1.68 × 10^-7^) and ileum (bottom, p = 4.7 × 10^-4^). Green shading indicates downregulation of the corresponding gene during lactation.

Thirty-one genes were identified as downregulated in all tissues (Additional File 6: Genes_with_decreased_mrna_all_tissues.doc). According to the over-representation analysis in DAVID, the KEGG T cell receptor pathway was over-represented in this group (p = 0.004) [29,30] (Additional File 5), although only three genes appeared in this list. This pathway did not pass any of the multiple testing procedures available in DAVID, but is consistent with the IPA results, which flagged "T-cell signaling and differentiation" to be downregulated in the jejunum and ileum (Figure 1).

Genes downregulated only in the duodenum were also investigated using DAVID, as several genes revealed a strong downregulation in this tissue (Additional File 5). Many of these genes have been identified as being expressed in the pancreas, i.e., eight of the 34 genes in this group matched the Sp_PIR keyword "pancreas" (p = 5.00 × 10^-15^). The function of these genes in the duodenum and the reason for their poor expression in lactation is not known.

The list of genes upregulated in all parts of the small intestine was not significantly enriched by any KEGG pathways in DAVID. The term Lipid Biosynthetic Process was overrepresented (p = 0.001), although this term did not pass any multiple testing correction available in DAVID.

"The Fibronectin Type III fold" Interpro entry was flagged as overrepresented in the list of genes downregulated in the small intestine, but not in liver [*Interleukin receptor 22, alpha 2 (Il22ra2)*, *Immunoglobulin superfamily 9 *(*Igsf9), Insulin receptor (Insr), Rims binding protein 2 (Rimbp2)*, and *Protein tyrosine phosphate receptor type g *(*Ptprg*)]. However, the relevance of the downregulation of these genes in lactation is not known.

Categories overexpressed in the list of genes upregulated in the liver only included the gene ontologies for "response to nutrient levels" and "cholesterol metabolic process". The list of genes downregulated in the liver showed the gene ontology associated with positive regulation of programmed cell death and may partially explain the increased liver size during lactation [31,32].

### Bile acid biosynthesis

Expression of *Cyp7a1*, the enzyme catalyzing the rate limiting step of bile acid biosynthesis, was detected to be increased (p = 0.0002) in the liver with a 1.76-fold change. Few other changes were detected in the bile acid biosynthetic pathway. Expression of *Cyp46a1, Ch25h, Cyp27a1, Cyp39a1, Cyp7b1, Cyp8b1, Akr1d1, Slc27a5, Acox2, Scp2*, and *Baat *[33] did not show a significant change in the liver, suggesting that the increase in bile acid biosynthesis observed was triggered by the increase in Cyp7a1 mRNA [15]. These data are consistent with our earlier detailed characterization of mRNA and protein expression of Cyp7a1, Cyp27a1 and Cyp8b1 in lactation [15].

### Ingenuity Pathways Analysis

The lists of differentially expressed genes for each tissue, based on the overall physiologic state effect p-value and the respective simple effects were examined by IPA. The three overrepresented pathways with the lowest p-values in each tissue are shown in Table 3, and selected pathways are discussed below.

**Table 3:** Top three pathways for overrepresentation in each tissue

Liver			
**Pathway**	**Fisher's Exact test p-value**	**BH p-value**	**Members**

Biosynthesis of steroids	3.50E-07	5.80E-05	↑Dhcr7, ↑Fdft1,↑Hmgcr,↑Idi1, ↑Lss, ↑Mvd, ↑Sqle

LXR/RXR Activation	1.57E-04	1.30E-02	↓Abcg5, ↓Abcg8, ↑Acaca, ↑Cyp7a1, ↓Hadh, ↑Hmgcr, ↓Lcat

Pentose/Phosphate Pathway	3.01E-04	1.67E-02	↑Aldoc, ↑G6pd, ↑Gpi, ↓H6pd, ↓Pgm5

			

**Duodenum**			

**Pathway**			

Biosynthesis of steroids	1.13E-10	1.74E-08	↑Dhcr7, ↑Fdft1, ↑Fntb, ↑Hmgcr, ↑Idi1, ↑Lss, ↑Mvd, ↑Sc5dl, ↑Sqle

Androgen and estrogen metabolism	1.35E-04	1.04E-02	↑Ftsj1, ↑Hsd11b1, ↑Hsd17b7, ↑Nsdhl, ↓Srd5a2, ↓Ugt2b7

Glycerolipid metabolism	1.46E-03	7.50E-02	↓Cel, ↓Clps, ↓Glb1l2, ↓Pnlip, ↓Pnliprp1, ↓Pnliprp2

			

**Jejunum**			

**Pathway**			

T-Cell receptor signaling	1.68E-07	2.78E-05	↓Bmx, ↓Cd8b, ↓Grap2, ↓Itk, ↓Malt1, ↓Pik3cg, ↓Pik3r1, ↓Ppp3cc, ↓Prkcq, ↓Ptprc, ↓Rasgrp1, ↓Trb

CD28 signaling in T helper cells	1.17E-05	6.74E-04	↓Grap2, ↓Itk, ↓Malt1, ↑Mapk9, ↓Pik3cg, ↓Pik3r1, ↓Ppp3cc, ↓Prkcq, ↓Ptprc, ↓Trb

TR/RXR activation	1.23E-05	6.74E-04	↑Fga, ↑Gh1, ↓Klf9, ↑Ldlr, ↓Pik3cg, ↓Pik3r1, ↓Thra, ↓Thrb, ↓Thrsp

			

**Ileum**			

**Pathway**			

Biosynthesis of steroids	9.42E-08	1.71E-05	↑Cyp24a1, ↑Dhcr7, ↑Fdft1, ↑Hmgcr, ↑Idi1, ↑Mvd, ↑Sc5dl, ↑Sqle

TR/RXR activation	3.16E-07	2.86E-05	↑Fga, ↑Gh1, ↓Klf9, ↑Me1, ↓Nfcor2, ↓Pck1, ↓Pik3c2b, ↓Pik3cg, ↓Pik3r5, ↓Ppargc1a, ↓Thra, ↓Thrb

Thrombopoietin signaling	4.85E-05	2.53E-03	↓Irs2, ↓Pik3c2b, ↓Pik3cg, ↓Pik3r5, ↓Plcg1, ↓Plcg2, ↓Prkce, ↓Prkcq

The pathways with the three lowest p-values from Fisher's exact test for pathways from IPA's "Canonical Pathways" database for each tissue are reported here along with the corresponding p-value from a right-tailed Fisher's exact test, the Benjamini-Hochberg (BH) adjusted p-value, and the genes within the pathway that showed differential expression within the respective tissue (Members). Arrows in the Members column indicate the direction of change in lactating animals relative to controls.

### Cholesterol synthesis and metabolism

"Biosynthesis of steroids" had the lowest p-value among IPA's "Canonical Pathways" in three of the four tissues, with the jejunum being the exception. At the designated cutoff (p < 0.01), the jejunum showed a much more modest change in the "biosynthesis of steroids" pathway (p = 0.011 for overrepresentation in jejunum; p < 1 × 10^-6 ^in all other tissues). Detailed visualization of the pathway revealed that the upregulated sections of the "biosynthesis of steroids" pathway corresponded with cholesterol synthesis (Additional Files 7, 8, 9, 10: Biosynthesis_of_sterols_in_liver.jpg, Biosynthesis_of_sterols_in_duodenum.jpg, Biosynthesis_of_sterols_in_jejunum.jpg, and Biosynthesis_of_sterols_in_ileum.jpg). Since cholesterol synthesis is regulated by Srebp proteins, Srebp-regulated genes were investigated further. To determine if an exceptionally large number of Srebp-regulated genes were in the list of differentially expressed genes, a list of genes shown to be regulated by Srebp by detection through microarray analysis in Srebp-overexpressing and Scap knockout mice was used for a right-tailed Fisher's Exact test [34] using an online calculator (http://www.langsrud.com/fisher.htm). Here, a p-value of <0.01 in the tissue being tested was defined as a positive test for the purpose of determining whether a given gene was differentially expressed.

**Figure 2:**
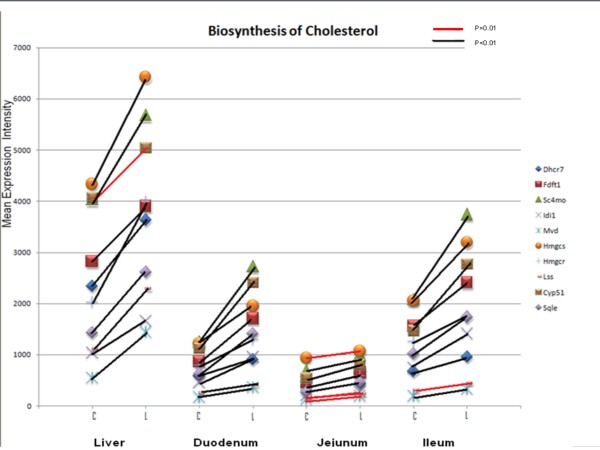
**Plot of Mean Intensities for Known Cholesterol Biosynthetic Enzymes**. The mean untransformed intensities were plotted against tissue/physiologic state combination. C and L indicate the mean of Control and Lactating samples, respectively, within each tissue. Abbreviations for genes are as follows. Dhcr7: 7-dehydrocholesterol reductase; Fdft1: farnesyl-diphosphate farnesyltransferase 1; Sc4mol: sterol-C4-methyl oxidase-like; Idi1: isopentenyl-diphosphate delta isomerase 1; Mvd: mevalonate (diphospho) decarboxylase; Hmgcs: 3-hydroxy-3-methylglutaryl-Coenzyme A synthase 1; Hmgcr: HMG Coenzyme A reductase; Lss: lanosterol synthase; Cyp51: cytochrome P450 family 51; Sqle: Squalene Epoxidase. A red line between Control and Lactating symbols for a given gene indicates p > .0.01 for the tissue pairwise comparison within the tissue from the mixed models repeated measures ANOVA on the log_2 _transformed intensities; the change was not considered significant for the purposes of determining a list of differentially expressed genes. A black line between C and L symbols indicates p < 0.01 as described above.

Of the 33 genes reported to be regulated by nuclear Srebp proteins [34], 29 were present in the data set. A Fisher's exact test p-value of p < 0.001 was calculated for each tissue, with the jejunum (p = 0.00029) having the fewest genes displaying a significant change (eight genes). Additional File 11: Genes_Regulated_by_Srebp_proteins.doc lists members of the cholesterol biosynthetic pathway and other genes that have been shown to be differentially expressed in Srebp-overexpressing mice and Scap knockout mice [34] and indicates the p-values for each tissue and the ratio of the background-corrected, normalized, untransformed intensities (lactation intensity/control intensity). As shown in Figure 2, Hmgcr mRNA expression was increased in three of the four tissues, with no change detected in the jejunum (p = 0.14). mRNA expression for the genes (*Srebf1, Srebf2*) encoding the Srebp proteins were not differentially expressed, although a tendency for a change was detected in the jejunum, where the simple effect comparison p-value for Srebf1 was 0.0006, and the overall physiologic state effect p value was 0.054. Expression of Insig1 mRNA, which is regulated by Srebp activity, showed a significant increase in each part of the small intestine. In contrast, an increase in Scap mRNA occurred in the liver only (p = 2.18E-7). Ldlr mRNA was upregulated in the duodenum and jejunum, and Tmem97 mRNA, an Srebp target [26], was upregulated in all tissues; both Ldlr and Tmem97 proteins aid in LDL uptake by cells [26].

### Cholesterol uptake

The only gene known to mediate cholesterol uptake in the gut, Npc1l1 [35], is not contained in the extended dataset for the Affymetrix Rat Exon 1.0ST. Investigation of the "full" and "all" datasets indicated that no probeset on the chip was annotated as Npc1l1. Therefore, expression of Npc1l1 was investigated by RT-PCR (Table 1). No significant changes were detected in Npc1l1 expression in any tissue. Abcg5 and Abcg8, which function as a heterodimer to efflux cholesterol from the enterocyte into the gut lumen and from the hepatocyte into bile [36], showed decreased expression in the liver (Abcg5 p = 2.9 × 10^-7^; Abcg8 p = 3.6 × 10^-6^) and in the ileum (Abcg5 p = 0.0062; Abcg8 p = 0.0048) (Table 4).

**Table 4:** ATP-Binding Cassette (ABC) transporters detected to be differentially expressed between Control and Lactating dams

Gene Symbol	Transcript Cluster ID	RL	RD	RJ	RIL	p < 0.01
Abca3	7065486	0.888 (p = 0.138)	0.931 (p = 0.329)	0.99 (p = 0.874)	0.8 (p = 0.007)	IL

Abca8a_predicted*	7083965	0.567 (p = 7.0E-04)	0.885 (p = 0.273)	0.942 (p = 0.744)	0.763 (p = 0.054)	L

Abcb1a	7250393	0.63 (p = 0.001)	0.663 (p = 0.002)	0.652 (p = 0.002)	0.697 (p = 0.005)	L,D,J,IL

Abcc5	7089622	0.818 (p = 0.021)	1.065 (p = 0.476)	0.938 (p = 0.471)	0.672 (p = 8E-05)	IL

Abcc6	7051110	0.886 (p = 0.109)	0.852 (p = 0.03)	0.884 (p = 0.089)	0.73 (p = 2.0E-04)	IL

Abcg2	7254219	0.949 (p = 0.666)	1.55 (p = 6.0E-05)	1.049 (p = 0.613)	1.203 (p = 0.049)	D

Abcg5	7303106	0.321 (p = 3.0E-07)	0.762 (p = 0.095)	0.901 (p = 0.515)	0.656 (p = 0.006)	L, IL

Abcg8	7294657	0.297 (p = 4.0E-06)	0.67 (p = 0.039)	0.803 (p = 0.261)	0.592 (p = 0.005)	L, IL

The p < 0.01 column indicates in which tissues a change was detected. Abbreviations are as defined for Table 1. *Gene is at the Extended level of confidence.

### Transporters

The (ABC) transporters that showed a significant change in at least one tissue were also investigated (Table 4). The ABC transporters are a superfamily of membrane transporters with diverse substrates that in eukaryotes mediate the ATP-dependent efflux of endogenous substrates, including bile acids and cholesterol, as well as of xenobiotics, including many drugs. The ABC transporter Abcb1a (Mdr1a) was downregulated in every tissue. This protein effluxes xenobiotics across the apical domain of the hepatocyte and enterocyte and plays an important role in limiting absorption of orally administered substrates [37]. Two members of the Abcc (Mrp) subfamily, Abcc5 and Abcc6, were downregulated in the ileum, while Abcg2 expression was increased in the duodenum.

Solute carrier proteins (Slcs) are a superfamily of proteins that transport many different molecules, including amino acids and ions (http://www.bioparadigms.org). All Slcs on the chip were investigated and those with a detected significant change in lactation in any tissue are shown in Additional File 12: Slcs.doc. For each tissue, nearly 20% of the Slcs showed a change at an overall physiologic state effect of p < 0.05, and approximately 5% were declared significant after applying a p < 0.01 cutoff within a tissue (simple effect)

Slc39a4 is a transporter mediating the uptake of zinc in the intestine [38]. *Slc39a4_predicted *was one of fifteen genes to be significantly upregulated in every tissue (p < 1 × 10^-5^) (Additional File 1). Fold changes for *Slc39a4 predicted *based on untransformed intensity values ranged from 1.65 in the jejunum to 2.81 in the liver (Table 2).

### Thyroid signaling

A Fisher's exact test using IPA detected significant overrepresentation in the TR/RXR pathway in every tissue. (Additional Files 13, 14, 15, 16: Liver_canonical_pathways.txt, Duodenum_canonical_pathways.txt., Jejunum_canonical_pathways.txt., and Ileum_canonical_pathways.txt). Thyroid hormone receptor α (TRA, Thra) and thyroid hormone receptor β (TRB, Thrb) were both downregulated in the ileum and jejunum. A decrease was also seen in the liver, but the change was not significant (p = 0.07 for TRA; p = 0.018 for TRB). Surprisingly few of the downstream genes of TR/RXR were downregulated in the IPA depiction of this pathway. In some cases, overlap occurred with Srebp signaling, and increased signaling from Srebp appeared to have overridden decreased thyroid signaling. This seems to have occurred with *Acetyl-CoA Carboxylase alpha (Acaca) *in the liver and *Ldlr *in both the duodenum and jejunum [34]. One TRB/RXR regulated gene [39], *Kruppel like factor 9 (Klf9)*, which is a transcription factor associated with intestinal proliferation, was downregulated in every tissue (Additional Files 17, 18, 19, 20: Liver_thyroid_pathway.jpg, Duodenum_thyroid_pathway.jpg, Jejunum_thyroid_pathway.jpg, and Ileum_thyroid_pathway.jpg). Klf9 knockout mice have shorter intestinal villi, although Klf9 is typically considered a transcriptional repressor and can also negatively regulate growth [40].

### Decreased mRNA from T-Cell receptor signaling and related pathways

mRNA of genes coding for the components of T-Cell receptor signaling pathway in IPA showed significant downregulation in the jejunum and ileum (p = 1.68 × 10^-7 ^and p = 4.7 × 10^-4^, respectively) (Figure 1). A similar pathway, the "CD28 receptor signaling in T helper cells" pathway was also downregulated in the jejunum, but substantial overlap between the two pathways suggested observation of the same events. These pathways are upstream of IL-2 production [41], however, the microarray detected no change in IL-2 mRNA in any tissue.

## Discussion

### Cholesterol Biosynthesis

IPA and DAVID both flagged "Biosynthesis of Steroids" to be overrepresented in the list of differentially expressed genes in lactation in three of the four tissues (the jejunum displayed a p-value near the cutoff), and the list of genes upregulated in all tissues during lactation, respectively (Additional Files 7, 8, 9, 10). Visualization of this pathway revealed that the genes identified were components of the cholesterol biosynthetic pathway. Statistical pattern matching as well as results from overrepresentation analyses in both DAVID and IPA indicated that expression of cholesterol biosynthetic genes was induced in all tissues examined, although to a lesser extent in the jejunum (p = 0.011; Benjamini-Hochberg corrected p = 0.048, values calculated by IPA). Cholesterol and lipid biosynthetic genes are known to be regulated by transcription factors known as the Srebp proteins. Three Srebp proteins are encoded by two genes, *Srebf1 *and *Srebf2. Srebf1 *codes for Srebp-1a and Srebp-1c, while *Srebf2 *codes for Srebp-2. The Srebp proteins differ in their control of fatty acid synthesis and cholesterol biosynthesis [27,42,43]. Srebp-2 is associated with cholesterol biosynthesis, while the Srebp1 proteins are associated with fatty acid synthesis [27], although there appears to be overlap in the genes that are responsive to these transcription factors [34]. Srebp-1c is sensitive at the transcriptional level to LXR signaling [42,44,45], while all three share a mechanism for becoming an active transcription factor [42]. Under conditions of sufficient cholesterol concentrations, the protein product of Insulin stimulated gene (Insig) binds to Srebf Chaperone (Scap) to retain an Insig/Scap/Srebp complex in the endoplasmic reticulum [42,46]. In the absence of oxysterols and cholesterol, Insig is degraded and Scap is released from the endoplasmic reticulum; Scap then escorts the bound Srebp to the Golgi, where the N-terminus of the Srebp is cleaved from the full protein to generate the active form that functions as a transcription factor [43]. As indicated above, cholesterol synthesis is increased during lactation in both the liver and the small intestine [2,3] and Srebp target genes have also been shown to be upregulated in mammary tissue in lactating dams [47].

The observation that Srebp-regulated genes were upregulated is consistent with early data showing an overall increased cholesterol biosynthesis in lactation [5]. We currently do not know which Srebp isoforms are involved in the changes seen in lactation, as Srebp-1c, Srebp-1a and Srebp-2, are all able to regulate expression of cholesterol synthetic genes. However, Srebp-2 plays a stronger role in regulating these genes [27]. The only potential change detected in the mRNA for a Srebf gene was *Srebf1*, the gene associated with Srebp-1a and Srebp-1c, which showed increased mRNA levels in the jejunum.

In the liver, Scap mRNA showed a significant increase (p = 2.2 × 10^-7^). If this change were associated with an increase in Scap protein levels, then a probable mechanism for the increase in Srebp target genes in the liver would be increased transport of Srebp proteins to the Golgi, and their subsequent delivery to the nucleus [42]. Insig1 mRNA also showed a significant increase in all parts of the small intestine, but not in the liver. Insig1 functions to retain Srebp in the endoplasmic reticulum. Thus, increased expression of Scap in liver and increased expression of Insig1 in intestine provide a likely mechanism for the greater increase in cholesterol synthesis in liver vs. intestine observed by Feingold et al [5].

A number of factors contribute to the increased need for cholesterol in the lactating dam. The dam requires significant cholesterol for the increased synthesis of bile acids; 50% of cholesterol catabolized in the liver from nonlactating rats is used for bile acid synthesis [48]. Since the size of bile acid pool increases 2-3-fold at 10 -14 d of lactation [15], greater than 50% of cholesterol is likely catabolized to bile acids in lactation. The proportion of dietary cholesterol vs. endogenously synthesized cholesterol that is catabolized to bile acids in nonlactating vs. lactating rats is not known. Most importantly, cholesterol is an essential component of milk that supports membrane synthesis and neurodevelopment in the pups [49]. About 16 mg per day of cholesterol is secreted into the milk in rats [50]; between 32 and 40% of this cholesterol is synthesized in the mammary gland, while 11% is absorbed from the diet [7]. Thus, cholesterol synthesized in the liver makes up about 50% of cholesterol secreted in milk [7].

In addition to detecting a change in cholesterol synthesis, a possible mechanism for improved net cholesterol uptake was found. *Abcg5 *and *Abcg8 *show decreased levels of mRNA expression in the liver and ileum. A decrease in the concentration of active Abcg5/Abcg8 heterodimer in the intestine would be expected to yield an increase in net cholesterol uptake through decreased efflux from the enterocyte into the gut lumen, while decreased hepatic expression would minimize cholesterol secretion into bile [51]. The decreased expression of Abcg5/g8 mRNA in the liver, together with increased expression of cholesterol synthetic genes, likely serve to enhance conservation of cholesterol to allow for sufficient transfer of cholesterol into the milk and for synthesis of bile acids. Increased synthesis of bile acids would in turn serve to increase cholesterol absorption [16]. Taken together, these data suggest a concerted mechanism for enhancing net cholesterol absorption and minimizing its elimination to ensure sufficient cholesterol for incorporation into milk and bile acid synthesis, both important factors in maintaining the health of both the dam and pups.

### Zinc

Statistical pattern matching found that Slc39a4_predicted mRNA was increased (1.65 to 2.81 fold) in all tissues. Slc39a4 is a major zinc transporter associated with zinc import into the enterocyte [38], and zinc absorption is up-regulated in lactation [52-55]. Taken together, these data imply that the increased expression of Slc39a4 mediates the increased zinc absorption that occurs in lactation. Zinc is an essential nutrient shown to be important in bone development [55], to play a role in stimulating the insulin pathway [56] and in controlling T-Cell activity [57]. Zinc requirements are increased during lactation relative to pregnancy, and therefore net zinc uptake needs to be increased to maintain zinc homeostasis during lactation in humans, particularly during early lactation [58]. Interestingly, alpha-2-macroglobulin (A2m) showed a substantial increase in mRNA expression (~15-fold) in the liver. Zinc can directly regulate A2m's ability to sequester cytokines [57,59,60] by enhancing formation of a form of A2m that contains free sulfhydryl groups, which serve as binding sites for the cytokines [60].

### Downregulation of mRNA from the T-Cell signaling pathway

Both the jejunum and ileum showed strong downregulation at the mRNA level of the proteins composing the T-Cell signaling pathway in IPA. These changes may reflect a decrease in the number of actual T-Cells in the small intestine of lactating rats.

### Xenobiotic Transporters

Table 4 displays significantly different changes in mRNA concentration for the ABC transporters. Included in this list of genes are *Abcb1a (Mdr1a), Abcc5, Abcc6, and Abcg2. Abcb1a *showed decreased expression in all tissues in the microarray and this was successfully validated for the jejunum and ileum by RT-PCR. *Abcc5 *and *Abcc6 *also showed decreased mRNA expression in the ileum. Decreased expression of these efflux transporters would in general lead to an increased net absorption of their substrates. In contrast, Abcg2 showed increased expression in the duodenum, which would decrease absorption of substrates. Sample substrates for these proteins include drugs such as digoxin and cyclosporine A (Abcb1a, [61]), cGMP (Abcc5 [62]), the glutathione conjugate leukotriene C_4 _(Abcc6 [63,64]), and 2-amino-1-methyl-6-phenylimidazo[4,5-b] pyridine, a dietary carcinogen (Abcg2 [65]). Further work is needed to understand the impact of these specific changes within the context of lactation on the lactating dam and her pups.

### TR/RXR Pathway

IPA found members of the TR/RXR pathway to be overrepresented in the list of differentially expressed genes in each tissue. In both the ileum and jejunum, both TRA and TRB showed down-regulation. Although lactating rats are hypothyroid [4] and expressed lower levels of mRNA for thyroid receptor in these tissues, not all thyroid responsive genes were down-regulated, including Apoa5, Eno1, and Glut1, which showed no change in any tissue. A more detailed picture of the thyroid receptor pathway can be found in Additional Files 17,18, 19, 20. Serum thyroid hormone levels and early steps in the pathway are likely downregulated as an attempt to conserve energy [4,66]. Fisher's exact test determines its p-values based on the counts of the number of genes in the list of differentially expressed genes and compares these to the total number of genes in the pathway relative to the total number of genes in the microarray. Therefore, pathways that overlap are likely to be detected as overrepresented if the overlapping genes are in the list of differentially expressed genes. Because some Srebp regulated genes are considered to be part of the TR/RXR pathway, the p-values for overrepresentation may be low, even if thyroid signaling overall was unchanged. However, changes in the mRNA levels of the thyroid receptors argue against this, since these receptors have not been shown to be Srebp targets.

## Conclusions

The present studies have shown an increase in the mRNA of enzymes involved in the cholesterol biosynthesis pathway, implying that the sterol regulatory element binding proteins are more active in the liver and small intestine in lactating vs nonlactating rats. The data are consistent with a coordinated response to the overall increased energy demands of lactation and the specific needs of the pups for cholesterol so that there is adequate cholesterol for incorporation into milk and increased synthesis of bile acids; the latter in turn function to increase the intestinal absorption of cholesterol and lipids. We also demonstrated a marked increase in the expression of a key transporter important in the uptake of the essential element, zinc. Finally, we detected decreased mRNA from genes associated with T-cell signaling in the jejunum and ileum.

## Methods

### Animals

Sixteen Sprague-Dawley rats that were lactating for 10-11 days and sixteen age-matched virgin controls were obtained from Harlan (Indianapolis, IN) and maintained on a 12 h light/dark cycle (6 AM lights on/6 PM lights off). Rats had free access to Teklad Global Diet 2018 (Harlan Laboratories, Cincinnati, OH) and water. In order to minimize the variance in energy demands on the lactating dam, pups were removed from large litters within 24 hours of birth so that all litters contained 8-11 pups. All animals were sacrificed at 16 h (10 h of light on a 12 h light/dark cycle; 4 PM), and the liver, duodenum, jejunum, and ileum were removed for total RNA extraction from each tissue. The first 5 cm of the small intestine following the pyloric sphincter was taken as the duodenum, while the 10 cm following the ligament of Trietz was discarded and the next 20 cm used as the jejunum. The 20 cm segment preceding the cecal valve was taken as ileum. The mucosal layer was removed by scraping at 4°C, and used for isolation of RNA from intestinal segments. RNA was extracted from homogenized tissue using Trizol (Invitrogen, Carlsbad, CA), and purified using RNeasy Mini Kit DNAse and columns (Qiagen, Valencia, CA). The integrity of all RNA samples was verified using an Agilent 2100 Bioanalyzer (Agilent Technologies, Santa Clara, CA). Animal protocols were conducted in accordance with the National Institutes of Health Guidelines for the Care and Use of Laboratory Animals and were approved by the Institutional Animal Care and Use Committee of the University of Kentucky.

Each rat was assigned to one of four pools within the respective physiologic state (four control pools and four lactating pools). Pooled RNA samples, consisting of the RNA from the four rats within the same group, were created for each tissue, with individual rats composing the pools consistent across tissues. Each pooled sample (RNA from one tissue from one set of four rats) was loaded onto a separate chip. This resulted in the use of 32 chips (4 tissues × 2 "physiologic states" × 4 pools). Samples were prepared and processed according to the manufacturer's instructions by the University of Kentucky Microarray Core Facility (Lexington, KY).

### Selection of Genes on Which to Perform Statistical Analysis

Affymetrix Expression Console software was used to perform the Robust Multichip Average (RMA) [67,68] algorithm, which background corrected, quantile normalized, and log_2_-transformed gene level summaries of the Extended dataset. Affymetrix has divided the chip into various datasets, which represent different confidence levels with respect to the complementarities between the probe and the sense strand of the gene sequences. The Extended dataset consists of the Core dataset, which is made up of Refseq entries and full length mRNAs, as well as additional multiple annotations based on cDNA libraries. Although the Core dataset probes are the best annotated, the rat genome is not as well annotated as the human and mouse genomes, with many of the genes in the rat Extended dataset identified based on their similarity to human or mouse genes. Use of the Extended dataset allowed a more thorough analysis of the genome. The summarized values are an average taken across all exons.

The exon level Affymetrix DABG (Detection Above Background) values and the Affymetrix annotation file (version raex_1_0-st-v1.na27.rn4) were used to filter the data [69]. Exon level data was opened in Expression Console and the RMA algorithm and log_2 _transformations were performed. An exon was considered present if it had a DABG p < 0.01, indicating that the exon in question had an intensity greater than 99% of the background probes with the same GC content [69]. A gene was considered for analysis if at least one exon was detected on at least two chips within the same tissue and the same physiologic state, e.g., presence on two control liver chips. Genes were also removed if the Affymetrix annotation file contained a "---" or a blank for the "mRNA description" entry (Annotation file: raex_1_0-st-v1.na27.rn4). Of the 19,434 genes in the Extended dataset, 14,129 were utilized for statistical tests based on these criteria.

### Statistical Analysis

Since all four tissues were taken from each rat, a repeated measures mixed model ANOVA was used to determine if changes in expression were statistically significant for each gene [70]. JMP genomics (SAS Institute Inc, Cary, NC) was used to perform the ANOVA using compound symmetry to model the covariance matrices. Tissue, physiologic state, and the tissue*physiologic state interaction were treated as fixed effects, while "pool", a variable describing the combination of four individual rats to create a sample, was treated as a random subject variable.

A common method for addressing multiple testing issues involved in the analysis of microarrays is the Benjamini-Hochberg false discovery rate (FDR) correction. Several possibilities existed for attempting to address the issue of multiple testing. First, the physiologic state p-value could be adjusted to a fixed FDR. Alternatively, the individual unadjusted p-values for the simple effects could be set to a given value and an approximation of the number of false positives within a group could then be calculated. We chose the latter approach, set p < 0.01 as a cutoff and calculated the proportion of false positives (PFPs) for the simple effects that represented the pairwise comparisons within a tissue. We chose this method to balance the risk of false positives with the risk of false negatives. False negatives in the list of differentially expressed genes could interfere with downstream pathway analyses.

The overall physiologic state effect of p < 0.05 also served as an additional cutoff to reduce the total number of statistical tests performed. Selection of an overall physiologic state p-value of p < 0.01 was problematic, as genes that displayed differential expression in only one tissue might not be noticed due to the lack of change in the remaining tissues. In summary, genes were considered differentially expressed between control and lactation within a tissue if a significant physiologic state effect was observed at p < 0.05 and a simple effect for the pairwise comparison within a tissue was p < 0.01.

.CEL files and .CHP files describing the data are available through GEO under GSE19175 and analyzed data is available in Additional File 1.

### Detection of Biological Trends

Ingenuity Pathways (IPA) (http://www.ingenuity.com) was used to screen the results for biological trends. Differentially expressed genes were determined as described in the "Statistical Analysis" section above. The Rat Exon 1.0 ST chip was used as a background list.

A right tailed Fisher's Exact test was used to screen the IPA database and detect categories that were overrepresented based on genes detected to have a significant difference in expression. Available in IPA is the Benjamini-Hochberg correction, which attempts to control the number of false positives. This calculation is dependent on the size of the database. We chose not to use the Benjamini-Hochberg correction, but chose to fix the significance threshold at p < 0.01 for any given test. A p < 0.01 for the Fisher's Exact test indicated that more genes in the list of differentially expressed genes appeared in a pathway than would be expected by chance if the same number of differentially expressed genes were to be selected randomly from all genes on the chip. Both these p-values and the Benjamini-Hochberg p-values are provided in Table 3.

The lists generated by statistical pattern matching were analyzed by DAVID [25,29] with the 14,129 genes used for statistical analyses as the background list.

The lists screened for overrepresentation were the Canonical Pathways category in IPA and several databases in DAVID (see below). For the Srebp transcription factors, a report of the differential gene expression in mice overexpressing isoforms of Srebp and in Scap knockout mice was used to create an additional list of genes known to be regulated by the Srebp proteins [34]. Out of the 33 genes listed as regulated by nuclear Srebp-1a and Srebp2, 29 were identified as being on the chip at the level of the Extended dataset and were used to perform a right tailed Fisher's Exact test.

We used IPA's Canonical Pathways database for all changes detected within a tissue, while we used all Gene Ontology terms, COG ontology, Sp_PIR Keyword [71,72], UP_SEQ_Feature [71,72], Interpro [73], PIR_Superfamily [74], SMART [75,76], and KEGG [30,77,78] as databases in DAVID for testing each pattern detected by statistical pattern matching.

### Statistical Pattern Matching

In order to assign changes of RNA expression into biologically meaningful groups, a method of statistical pattern matching was used analogous to the one used by Arzuaga et al [23] and Hulshizer and Blalock [24]. mRNA from any given gene could increase expression, decrease expression, or show no change in expression in samples from lactating animals compared to controls in each tissue. The method for pattern matching is described in a step by step manner as follows. Only genes that tested positive for differential expression were used for pattern matching. These genes were assigned an additional significance call at p < 0.05 for each simple effect within a tissue. This was done to protect against incorrectly assigning a gene that systematically had low p-values as differentially expressed in only one tissue. For example, if a gene was downregulated with p values of less than 0.01 in the liver, duodenum, and jejunum, and a p of 0.03 in the ileum, the gene was assigned as differentially expressed in all tissues rather than in three of the four tissues. Although the gene would not have been considered differentially expressed for the purposes of evaluating which genes were differentially expressed in the ileum, the gene was considered differentially expressed for the purposes of assigning a pattern. This reduced the risk of falsely assigning the gene to another pattern in the presence of a false negative. The fold-change within a given tissue was then utilized to determine if a gene was upregulated or downregulated in each tissue. For each tissue, three values were multiplied to define the change in that tissue. One value was an integer that represented the tissue itself: 1000 for liver, 100 for duodenum, 10 for jejunum, and 1 for ileum. The second value was a multiplier to define whether or not there was a tendency (p < 0.05) for the gene to change expression in the given tissue, where p < 0.05 = 1; p > 0.05 = 0). The third value defined the directionality of the change, where upregulation = 1 and downregulation = - 1. The products of these values were used to define the change that occurred within a given tissue. For example a gene upregulated in the liver would have a value of 1000, i.e., 1000*1*1. The sum of this value for all tissues was taken to generate a unique value for every set of possible changes that could occur across tissues. For example a gene that was upregulated in every tissue except the ileum, where it was downregulated would have a value of 1009 (1000 + 100 + 10 - 1). The following groups were defined to be of particular importance and were investigated: up in all tissues, down in all tissues, up in all parts of small intestine, down in all parts of small intestine, up in liver, down in liver, and down in duodenum only.

### RT-PCR validation of microarray findings

The same total RNA samples used for the microarray were used for RT-PCR validation; RT-PCR was performed as reported previously [79], using the geometric average of Ctsb, Tmbim6, and Tmed2 as a normalization constant [15]. An aliquot of all cDNA samples was used to generate a standard curve.

Choosing an appropriate normalization constant was performed using methodology similar to that described by Andersen et al [80]. Methods for detecting valid internal controls require a list of candidate genes with little or no bias, since any bias that exists within the genes detected as valid internal controls would be transferred over to the newly calculated normalization factor [80]. Genes with high expression (mean log_2 _intensity from the microarray greater than or equal to 10) were sorted by their coefficients of variance. Microarray data for the twenty genes with the lowest coefficients of variance were input into Normfinder [80]. The single best gene to use as a control was Ctsb, and the best combination of two was found to be Tmbim6 and Tmed2. We performed RT-PCR on these genes and chose to use the geometric mean of the three genes as a normalization constant. The same statistical model that was used to analyze the microarray results was used to analyze the normalized RT-PCR data.

cDNA was synthesized using High-Capacity cDNA Reverse Transcription Kits from Applied Biosystems (Foster city, CA) according to the manufacturer's instructions. Primers and Universal Probes Library (UPL) probes for real time RT PCR were designed and ordered from Roche Applied Science (Mannheim, Germany) using online software (http://www.universalprobelibrary.com). A list of primers is provided in Additional File 21: RT_PCR_primers.doc. The Roche Light Cycler 480 was used for performing the RT-PCR. Briefly, 1 μg of total RNA was used for cDNA synthesis, the synthesized cDNA diluted to 500 μL, and 5 μL of diluted cDNA used as template in a 20 μL reaction volume. For quantification analysis of real time RT-PCR data, a standard curve was generated by pooling all the cDNA samples to form one cDNA mixture and then diluting this cDNA mixture 10-, 100-, 1000- and 10,000-fold so that expression of the gene of interest was within the range of the standard curve.

## Authors' contributions

ATA performed statistical analyses and drafted the manuscript. LH performed statistical analyses. CRW, TZ, and PJ were involved in experimental design, sample collection and preparation. TZ also performed RT-PCR validation. AJS oversaw statistical analyses and aided in experimental design. MV led the experimental design, revised the manuscript, and oversaw the project. All authors read and approved the final manuscript.

## Supplementary Material

Additional File 1 **Statistical analysis and statistical pattern matching results (Statistical_Analysis_and_Statistical_Pattern_Matching_Results.txt)**. Results from statistical pattern matching are reported as a .txt file. Results are reported for all 14,129 genes considered for statistical analysis, but genes that were not differentially expressed in any tissue are assigned a pattern of zero. Transcript_ID is an identifier associated with the gene analyzed. The gene assignment entry was taken from the Affymetrix annotation file and displays which gene is associated with the given transcript ID. Tissue p, physiological state p, and tissue*physiological state p correspond to the p-values associated with the tissue main effect, physiological state main effect, and the interaction, respectively. p Liver, p duodenum, p jejunum, and p ileum represent the p-values associated with the corresponding simple effects Pattern indicates in which pattern a gene is detected. A zero indicates no differential expression. Otherwise, patterns are assigned as described in the Methods. Ratio liver, ratio duodenum, ratio jejunum, and ratio ileum represent the ratio of the mean of samples from lactating animals to the mean of samples from controls utilizing the untransformed microarray data. Click here for file

Additional File 2 **Benjamini-Hochberg false discovery rates (Benjamini_Hochberg_False_Discovery_Rates.txt)**. False Discovery Rate corrections [81] for all genes studied is presented in .txt format. Transcript_ID is an Affymetrix Identifier for each gene. False discovery rates are provided for the tissue*physiological state interaction term and the overall physiological state effect. Q-values were calculated as the number of false positives expected by chance (uncorrected p* total number of tests) divided by the total number of results with an equal or lower p-value. Click here for file

Additional File 3 **Histograms of p-values (Histograms_of_p_values.ppt)**. Histograms for A) tissue effect p-values, B) physiological state effect p-values, C) physiological state*tissue interaction p-values, and pairwise comparison p-values for D) the liver, E) duodenum, F) jejunum, and G) ileum presented as a .ppt file. While a large tissue effect was observed, a visible treatment effect (control vs. lactation) was also observed. Click here for file

Additional File 4 **Volcano plots (Volcano_plots.doc)**. Volcano plots comparing the log_2 _fold changes (reported as mean untransformed lactating intensity divided by untransformed mean control intensity) against the calculated pairwise comparison p-value for each individual tissue in .doc format. Volcano plots are for A) Liver, B) Duodenum, C) Jejunum, and D) Ileum. Each tissue responded differently to lactation. The blue line indicates the significance cutoff of p < 0.01. The number of differentially expressed genes were 420 in the liver, 337 in the duodenum, 402 in the jejunum, and 523 in the ileum, when an overall treatment main effect p-value cutoff of p < 0.05 was incorporated. Of particular note is a series of genes that were strongly downregulated in the duodenum (Additional File 1 pattern -100; discussed in Results.) Click here for file

Additional File 5 **DAVID output (DAVID_output_file.txt)**. DAVID was utilized to test specific patterns from statistical pattern matching. Patterns were chosen based on similarity between tissues and were "Up in All Tissues", "Down in all tissues", "Up in all parts of small intestine", "Down in all parts of small intestine", "Up only in liver", "Down only in liver", and "Down only in duodenum". Table truncated from output in DAVID. Genes listed by Affymetrix transcript cluster ID, which may be referenced in Additional File 1. Click here for file

Additional File 6 **Genes with decreased mRNA in all tissues (Genes_with_decreased_mrna_all_tissues.doc)**. To be considered part of a grouping, genes must have a physiologic state p < 0.05 and at least one tissue simple effect p < 0.01. Reported p-values are tissue simple effect p-values and represent the comparison between Lactation and Control in the corresponding tissue. For the purposes of assigning patterns, the significance cutoff for the remaining tissue simple effect was set to p < 0.05. Abbreviations used as in Table 1. *Gene is at the Extended confidence level. Click here for file

Additional File 7 **Biosynthesis of sterols in liver (Biosynthesis_of_sterols_in_liver.jpg)**. Image from IPA representing the "Biosynthesis of Sterols" in the liver as a .jpg file. Numbering system for enzymes in the pathway is taken from KEGG [77]. Components of the cholesterol biosynthetic pathway include 1.1.1.34 (Hmgcr), 2.7.1.36 (Mvk), 2.7.4.2 (Pmvk), 4.1.1.83 (Mvd), 5.3.3.2 (Idi1), 2.5.1.21 (Fdft1), and 1.14.99.7 (Sqle), 5.4.99.7 (Lss), and 1.3.1.21 (Dhcr7). Red shading indicates increased mRNA during lactation from the corresponding gene. Click here for file

Additional File 8 **Biosynthesis of sterols in duodenum (Biosynthesis_of_sterols_in_duodenum.jpg)**. Image from IPA representing the "Biosynthesis of Sterols" in the duodenum as a .jpg file. Numbering system for enzymes in the pathway is taken from KEGG [77]. Components of the cholesterol biosynthetic pathway include 1.1.1.34 (Hmgcr), 2.7.1.36 (Mvk), 2.7.4.2 (Pmvk), 4.1.1.83 (Mvd), 5.3.3.2 (Idi1), 2.5.1.21 (Fdft1), and 1.14.99.7 (Sqle), 5.4.99.7 (Lss), and 1.3.1.21 (Dhcr7). Red shading indicates increased mRNA during lactation from the corresponding gene. Click here for file

Additional File 9 **Biosynthesis of sterols in jejunum (Biosynthesis_of_sterols_in_jejunum.jpg)**. Image from IPA representing the "Biosynthesis of Sterols" in the jejunum as a .jpg file. Numbering system for enzymes in the pathway is taken from KEGG [77]. Components of the cholesterol biosynthetic pathway include 1.1.1.34 (Hmgcr), 2.7.1.36 (Mvk), 2.7.4.2 (Pmvk), 4.1.1.83 (Mvd), 5.3.3.2 (Idi1), 2.5.1.21 (Fdft1), and 1.14.99.7 (Sqle), 5.4.99.7 (Lss), and 1.3.1.21 (Dhcr7). Red shading indicates increased mRNA during lactation from the corresponding gene. Click here for file

Additional File 10 **Biosynthesis of sterols in ileum (Biosynthesis_of_sterols_in_ileum.jpg)**. Image from IPA representing the "Biosynthesis of Sterols" in the ileum as a .jpg file. Numbering system for enzymes in the pathway is taken from KEGG [77]. Components of the cholesterol biosynthetic pathway include 1.1.1.34 (Hmgcr), 2.7.1.36 (Mvk), 2.7.4.2 (Pmvk), 4.1.1.83 (Mvd), 5.3.3.2 (Idi1), 2.5.1.21 (Fdft1), and 1.14.99.7 (Sqle), 5.4.99.7 (Lss), and 1.3.1.21 (Dhcr7). Red shading indicates increased mRNA during lactation from the corresponding gene. Click here for file

Additonal File 11 **Genes regulated by Srebp proteins (Genes_Regulated_by_Srebp_proteins.doc)**. Genes that increase expression in Srebp-1a overexpressing mice and Srebp-2 overexpressing mice, and decrease expression in Scap knockout mice [34]. Overrepresentation analysis showed that genes in this list occurred more frequently than expected by chance in the lists of differentially expressed genes (p < 1 × 10^-4 ^in each tissue.) Abbreviations used as in Table 1. *Gene is at the Extended confidence level. Click here for file

Additional File 12 **Members of the Slc superfamily (Slcs.doc)**. Table displaying members of the Slc superfamily. The p < 0.01 column indicates in which tissues a change was detected. Abbreviations are as defined for Table 1. * Gene is at the Extended level of confidence. ^a^Substrates taken from the SLC tables database (http://www.bioparadigms.org/) Click here for file

Additional File 13 **Canonical pathways in the liver (Liver_canonical_pathways.txt)**. This file contains the canonical pathways in IPA and the corresponding -log_10 _p-values and the individual molecules that were detected as being in the list of overrepresented genes and part of each pathway (listed in the "molecules" column.) FDRs for the individual pathways are shown in a separate series of rows underneath the -log_10 _p-values. This table shows results utilizing the list of differentially expressed genes in the liver. Click here for file

Additional File 14 **Canonical pathways in the duodenum (Duodenum_canonical_pathways.txt)**. This file contains the canonical pathways in IPA and the corresponding -log_10 _p-values and the individual molecules that were detected as being in the list of overrepresented genes and part of each pathway (listed in the "molecules" column.) FDRs for the individual pathways are shown in a separate series of rows underneath the -log_10 _p-values. This table shows results utilizing the list of differentially expressed genes in the duodenum. Click here for file

Additional File 15 **Canonical pathways in the jejunum (Jejunum_canonical_pathways.txt)**. This file contains the canonical pathways in IPA and the corresponding -log_10 _p-values and the individual molecules that were detected as being in the list of overrepresented genes and part of each pathway (listed in the "molecules" column.) FDRs for the individual pathways are shown in a separate series of rows underneath the -log_10 _p-values. This table shows results utilizing the list of differentially expressed genes in the jejunum. Click here for file

Additional File 16 **Canonical pathways in the ileum (Ileum_canonical_pathways.txt)**. This file contains the canonical pathways in IPA and the corresponding -log_10 _p-values and the individual molecules that were detected as being in the list of overrepresented genes and part of each pathway (listed in the "molecules" column.) FDRs for the individual pathways are shown in a separate series of rows underneath the -log_10 _p-values. This table shows results utilizing the list of differentially expressed genes in the ileum. Click here for file

Additional File 17 **Thyroid pathway in liver (Liver_thyroid_pathway.jpg)**. Images from IPA for the TR/RXR pathway for the liver. Red shading indicates increased mRNA amounts of the respective gene during lactation, and green shading indicates decreased amounts of mRNA. Click here for file

Additional File 18 **Thyroid pathway in duodenum (Duodenum_thyroid_pathway.jpg)**. Images from IPA for the TR/RXR pathway for the duodenum. Red shading indicates increased mRNA amounts of the respective gene during lactation, and green shading indicates decreased amounts of mRNA. Click here for file

Additional File 19 **Thyroid pathway in jejunum (Jejunum_thyroid_pathway.jpg)**. Images from IPA for the TR/RXR pathway for the jejunum. Red shading indicates increased mRNA amounts of the respective gene during lactation, and green shading indicates decreased amounts of mRNA. Click here for file

Additional File 20 **Thyroid pathway in ileum (Ileum_thyroid_pathway.jpg)**. Images from IPA for the TR/RXR pathway for the ileum. Red shading indicates increased mRNA amounts of the respective gene during lactation, and green shading indicates decreased amounts of mRNA. Click here for file

Additional File 21 **RT-PCR primers (RT_PCR_primers.doc)**. Primer sequences for all genes analyzed by RT-PCR. Click here for file
