# Supplementary figures and images for: Differential gene expression in liver and small intestine from lactating rats compared to age-matched virgin controls detects increased mRNA of cholesterol biosynthetic genes
Source: BMC Genomics. 2011 Feb 3;12:95. doi: 10.1186/1471-2164-12-95 (PMC3045338; doi:10.1186/1471-2164-12-95)

## Slide 1
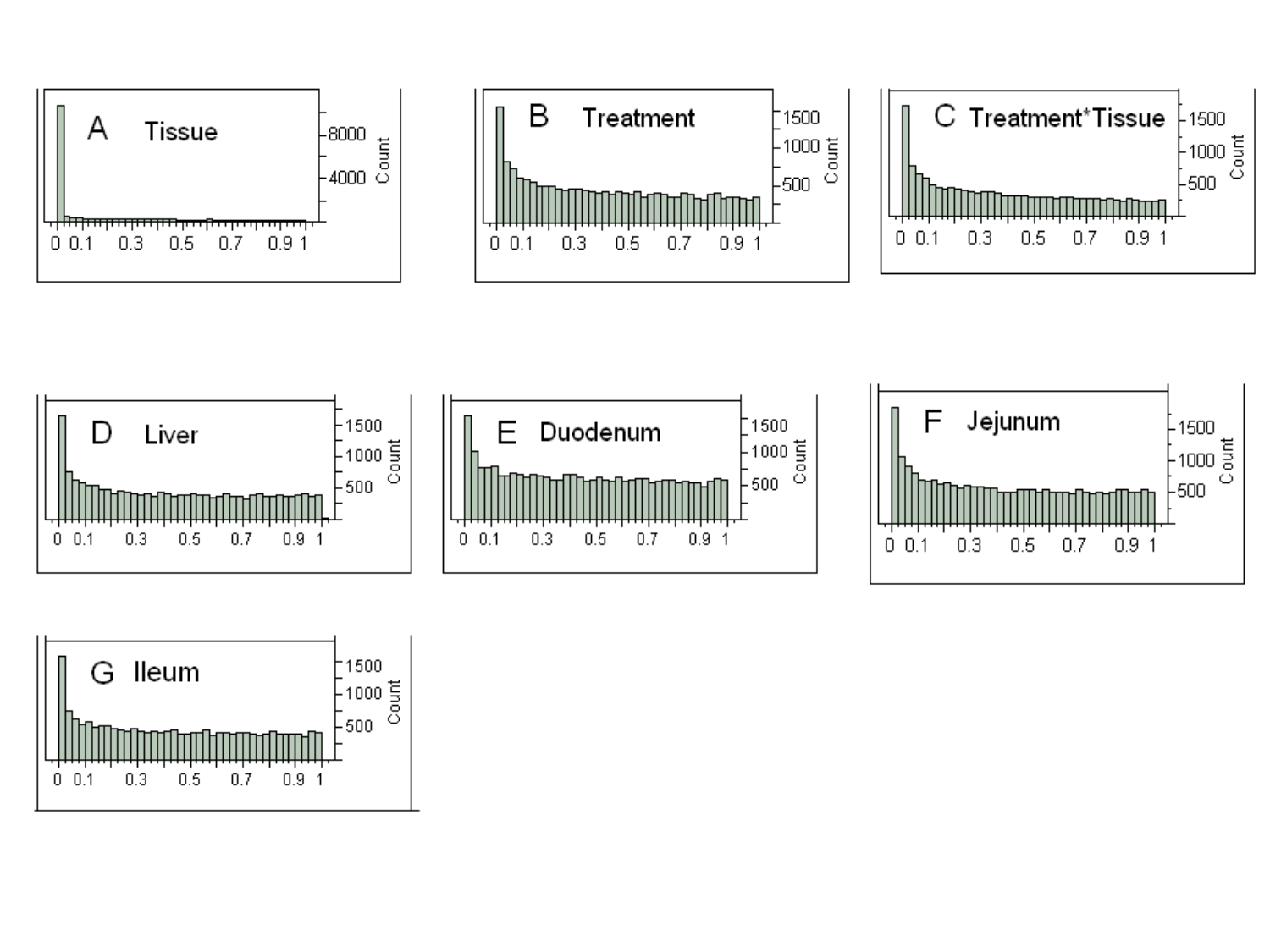

Supplement: Additional File 3 — Histograms of p-values (Histograms_of_p_values.ppt). Histograms for A) tissue effect p-values, B) physiological state effect p-values, C) physiological state*tissue interaction p-values, and pairwise comparison p-values for D) the liver, E) duodenum, F) jejunum, and G) ileum presented as a .ppt file. While a large tissue effect was observed, a visible treatment effect (control vs. lactation) was also observed. [file 1471-2164-12-95-S3.PPT]

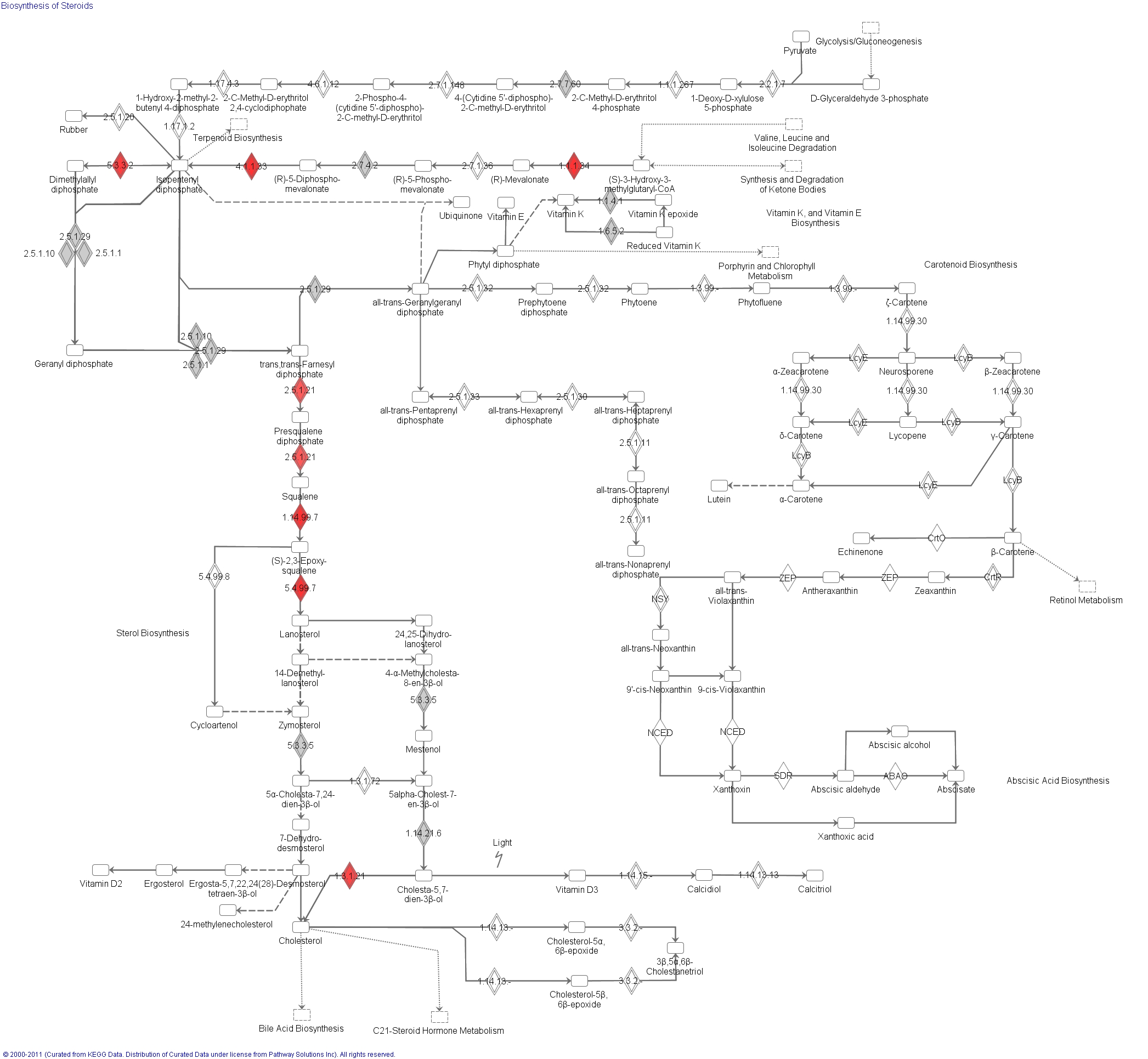

Supplement: Additional File 7 — Biosynthesis of sterols in liver (Biosynthesis_of_sterols_in_liver.jpg). Image from IPA representing the "Biosynthesis of Sterols" in the liver as a .jpg file. Numbering system for enzymes in the pathway is taken from KEGG [77]. Components of the cholesterol biosynthetic pathway include 1.1.1.34 (Hmgcr), 2.7.1.36 (Mvk), 2.7.4.2 (Pmvk), 4.1.1.83 (Mvd), 5.3.3.2 (Idi1), 2.5.1.21 (Fdft1), and 1.14.99.7 (Sqle), 5.4.99.7 (Lss), and 1.3.1.21 (Dhcr7). Red shading indicates increased mRNA during lactation from the corresponding gene. [file 1471-2164-12-95-S7.JPEG]

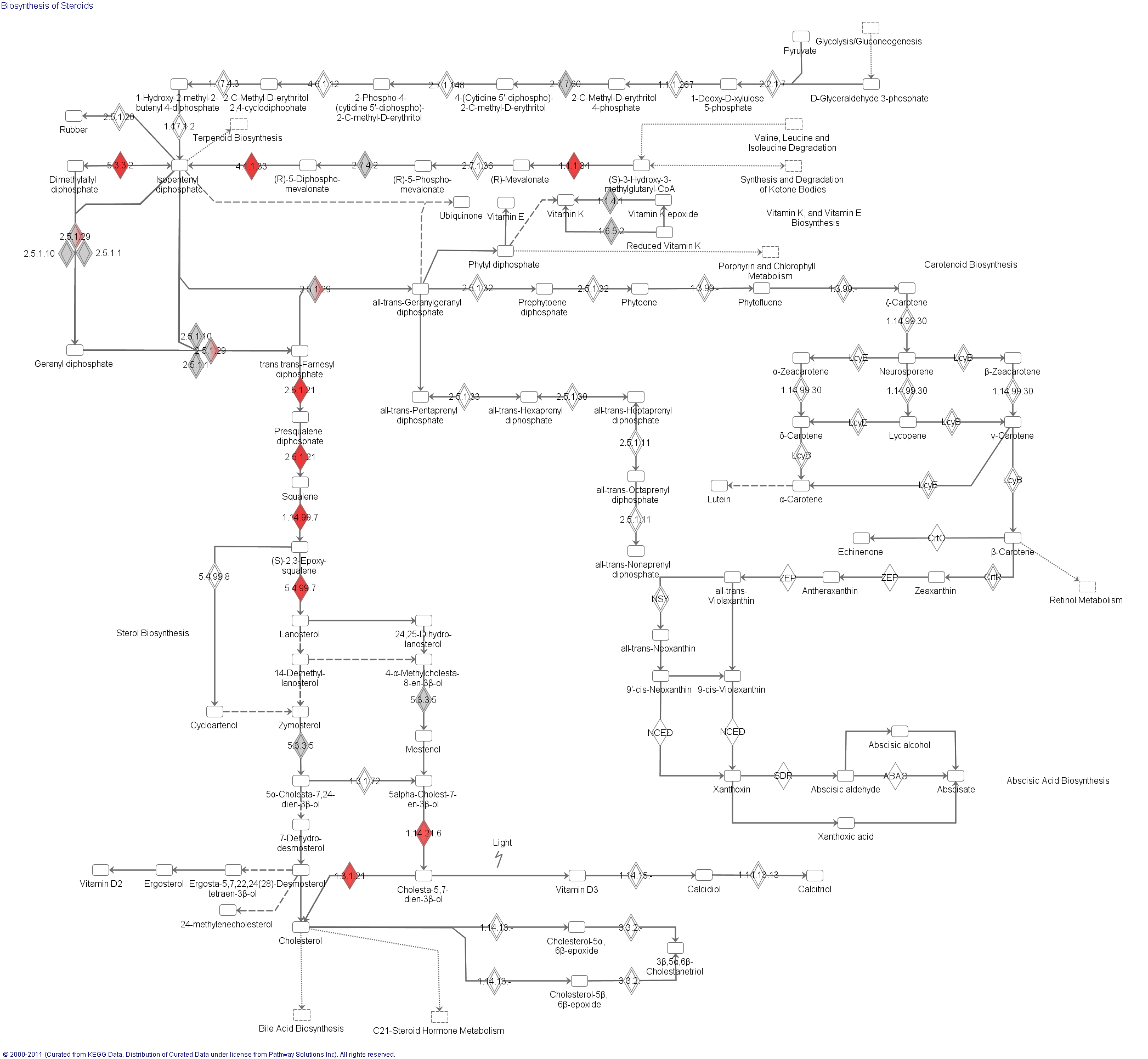

Supplement: Additional File 8 — Biosynthesis of sterols in duodenum (Biosynthesis_of_sterols_in_duodenum.jpg). Image from IPA representing the "Biosynthesis of Sterols" in the duodenum as a .jpg file. Numbering system for enzymes in the pathway is taken from KEGG [77]. Components of the cholesterol biosynthetic pathway include 1.1.1.34 (Hmgcr), 2.7.1.36 (Mvk), 2.7.4.2 (Pmvk), 4.1.1.83 (Mvd), 5.3.3.2 (Idi1), 2.5.1.21 (Fdft1), and 1.14.99.7 (Sqle), 5.4.99.7 (Lss), and 1.3.1.21 (Dhcr7). Red shading indicates increased mRNA during lactation from the corresponding gene. [file 1471-2164-12-95-S8.JPEG]

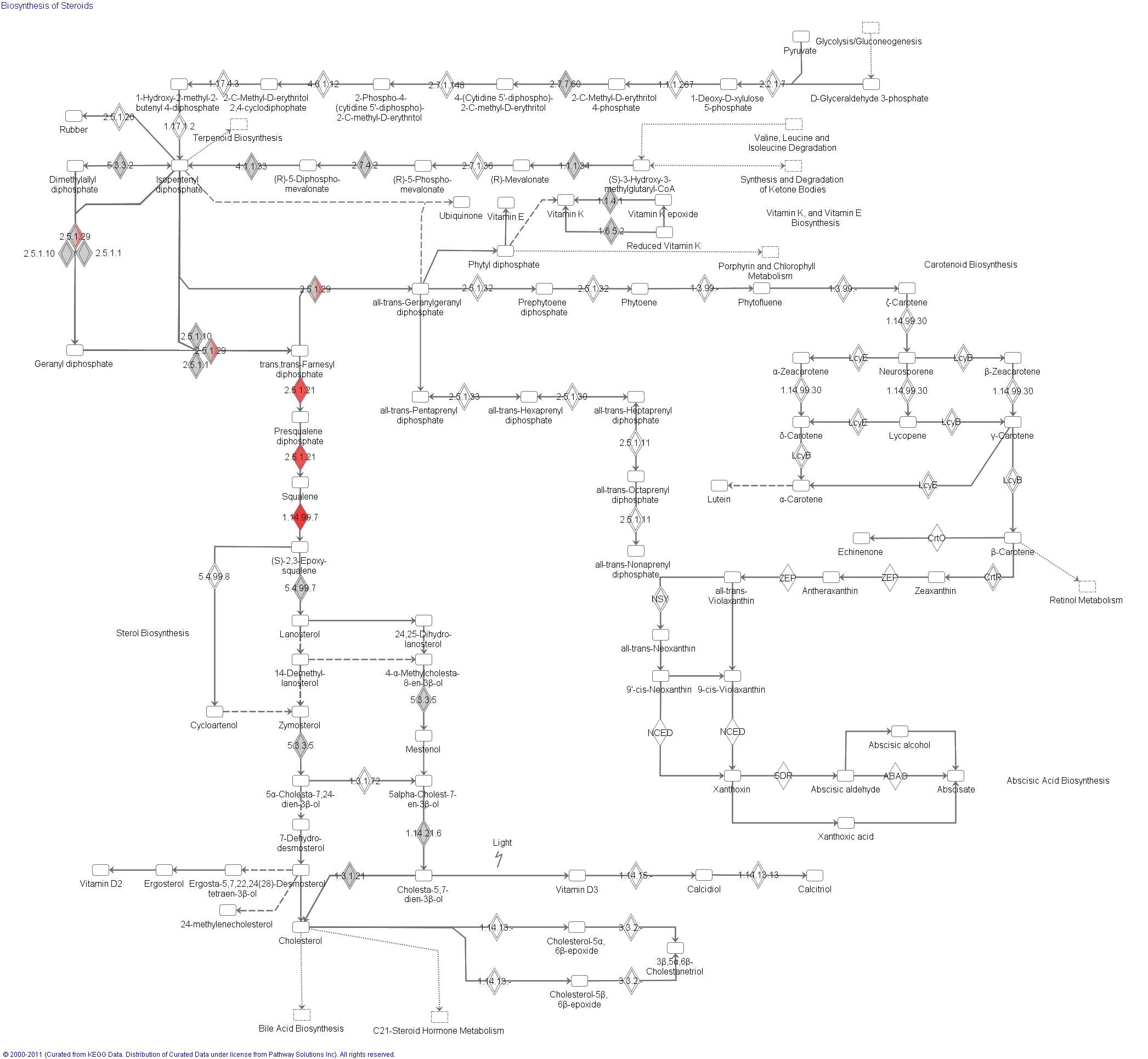

Supplement: Additional File 9 — Biosynthesis of sterols in jejunum (Biosynthesis_of_sterols_in_jejunum.jpg). Image from IPA representing the "Biosynthesis of Sterols" in the jejunum as a .jpg file. Numbering system for enzymes in the pathway is taken from KEGG [77]. Components of the cholesterol biosynthetic pathway include 1.1.1.34 (Hmgcr), 2.7.1.36 (Mvk), 2.7.4.2 (Pmvk), 4.1.1.83 (Mvd), 5.3.3.2 (Idi1), 2.5.1.21 (Fdft1), and 1.14.99.7 (Sqle), 5.4.99.7 (Lss), and 1.3.1.21 (Dhcr7). Red shading indicates increased mRNA during lactation from the corresponding gene. [file 1471-2164-12-95-S9.JPEG]

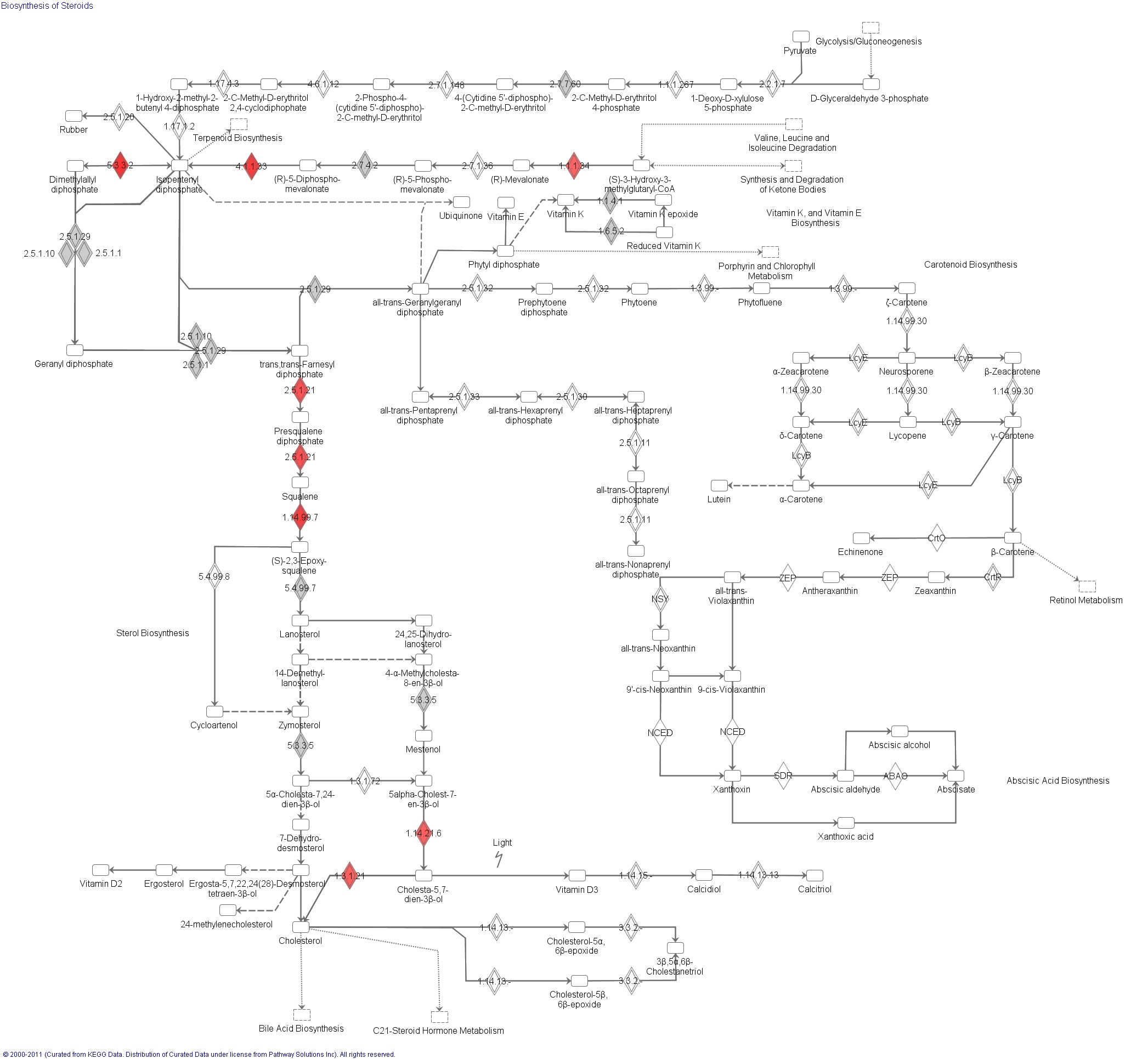

Supplement: Additional File 10 — Biosynthesis of sterols in ileum (Biosynthesis_of_sterols_in_ileum.jpg). Image from IPA representing the "Biosynthesis of Sterols" in the ileum as a .jpg file. Numbering system for enzymes in the pathway is taken from KEGG [77]. Components of the cholesterol biosynthetic pathway include 1.1.1.34 (Hmgcr), 2.7.1.36 (Mvk), 2.7.4.2 (Pmvk), 4.1.1.83 (Mvd), 5.3.3.2 (Idi1), 2.5.1.21 (Fdft1), and 1.14.99.7 (Sqle), 5.4.99.7 (Lss), and 1.3.1.21 (Dhcr7). Red shading indicates increased mRNA during lactation from the corresponding gene. [file 1471-2164-12-95-S10.JPEG]

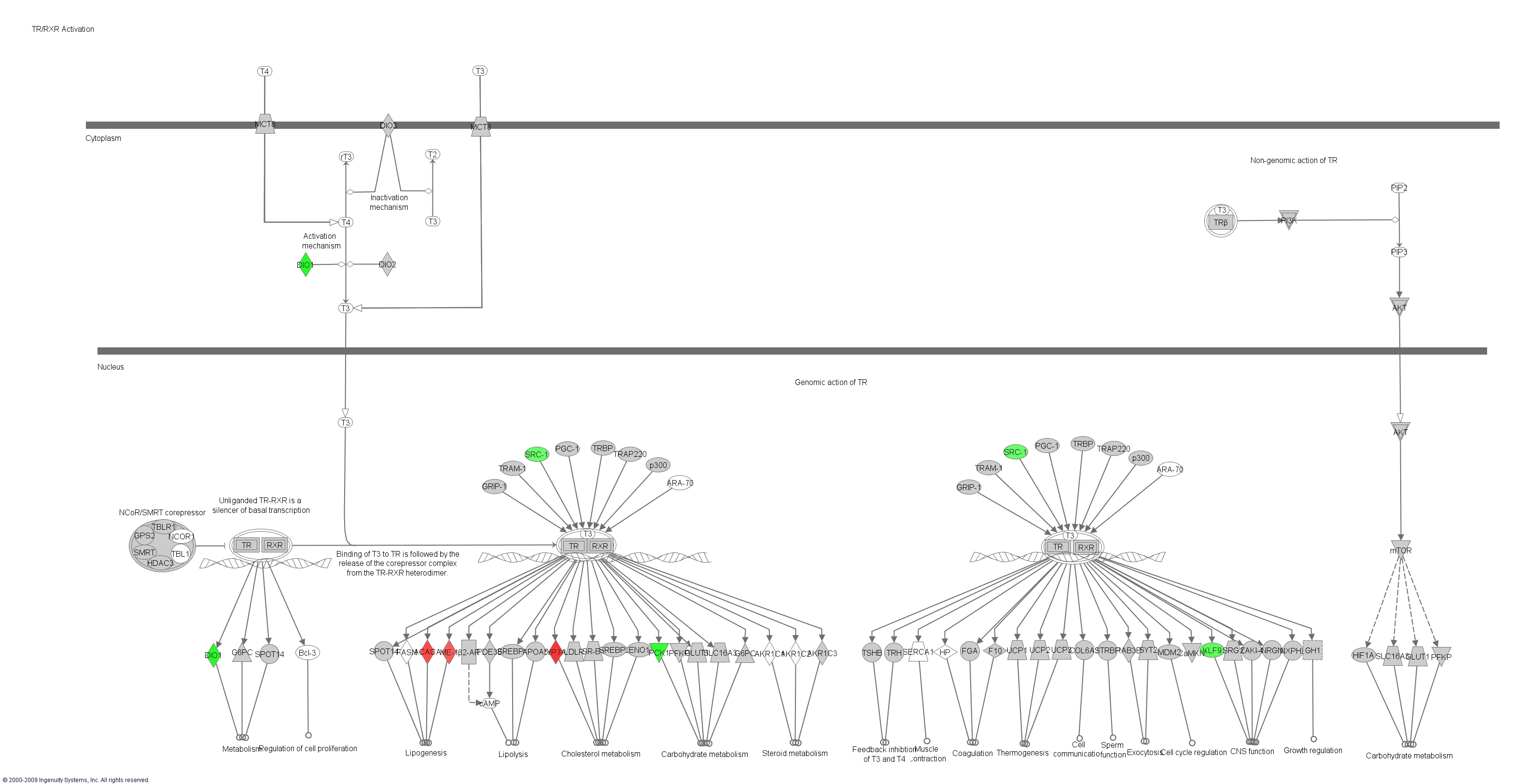

Supplement: Additional File 17 — Thyroid pathway in liver (Liver_thyroid_pathway.jpg). Images from IPA for the TR/RXR pathway for the liver. Red shading indicates increased mRNA amounts of the respective gene during lactation, and green shading indicates decreased amounts of mRNA. [file 1471-2164-12-95-S17.JPEG]

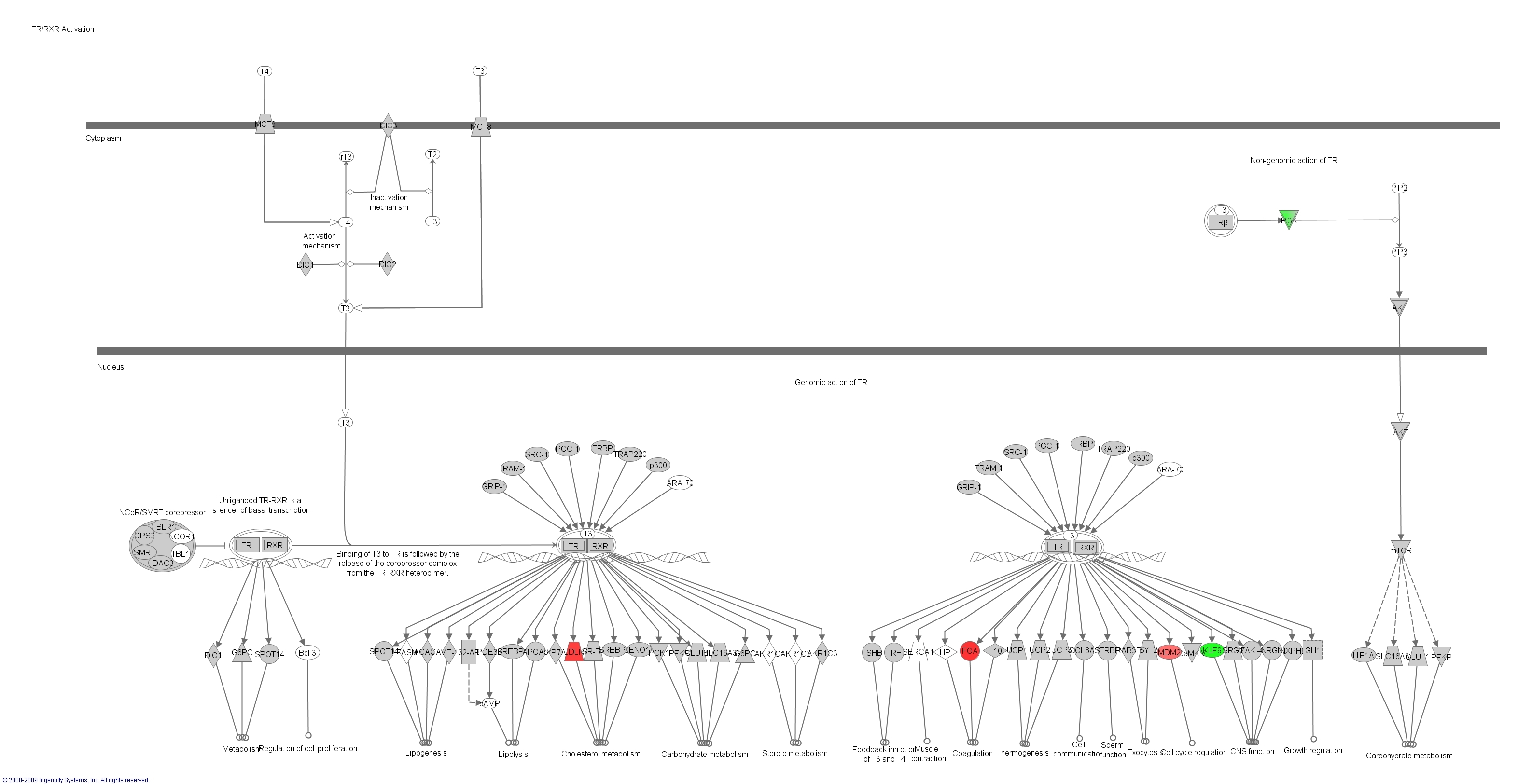

Supplement: Additional File 18 — Thyroid pathway in duodenum (Duodenum_thyroid_pathway.jpg). Images from IPA for the TR/RXR pathway for the duodenum. Red shading indicates increased mRNA amounts of the respective gene during lactation, and green shading indicates decreased amounts of mRNA. [file 1471-2164-12-95-S18.JPEG]

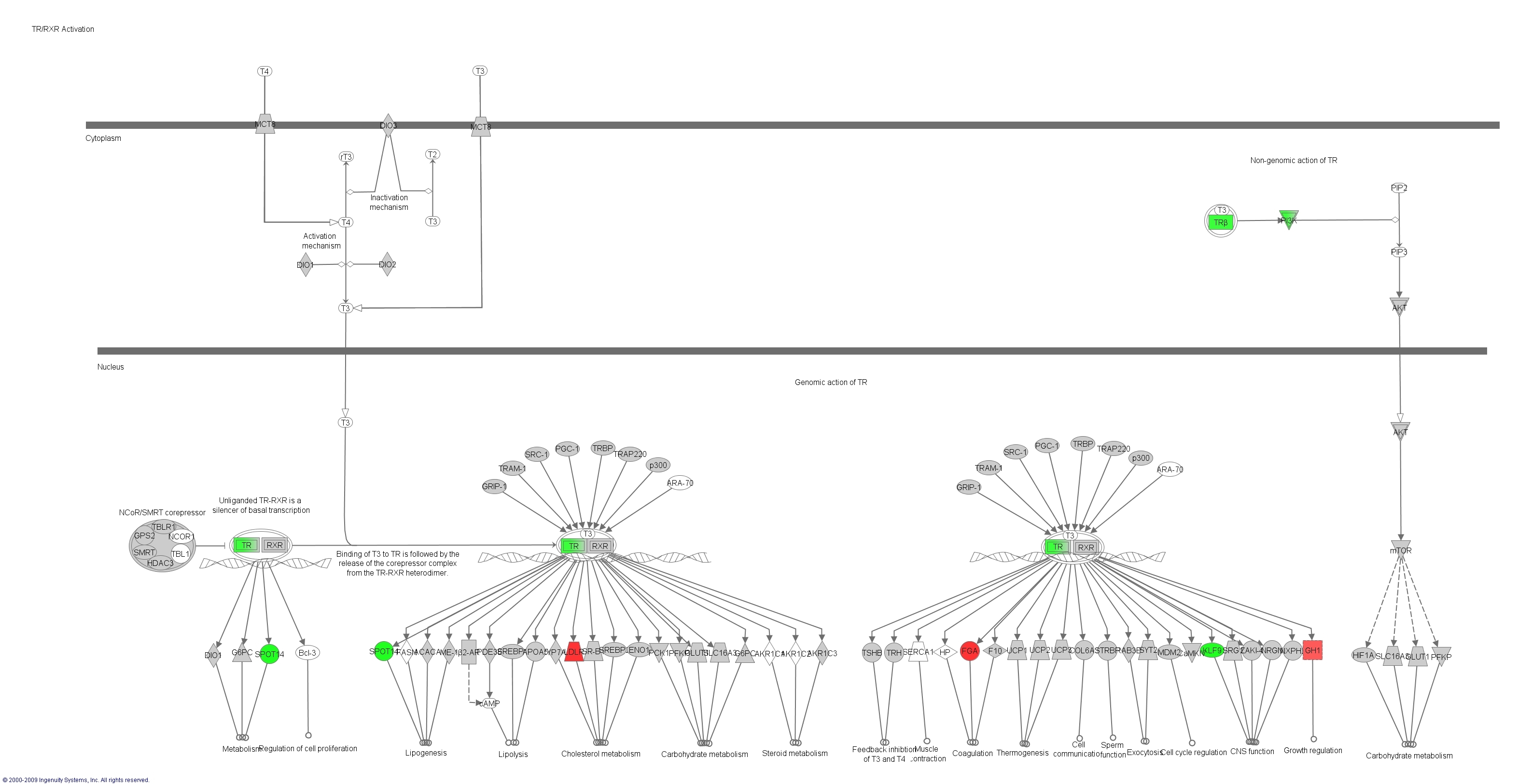

Supplement: Additional File 19 — Thyroid pathway in jejunum (Jejunum_thyroid_pathway.jpg). Images from IPA for the TR/RXR pathway for the jejunum. Red shading indicates increased mRNA amounts of the respective gene during lactation, and green shading indicates decreased amounts of mRNA. [file 1471-2164-12-95-S19.JPEG]

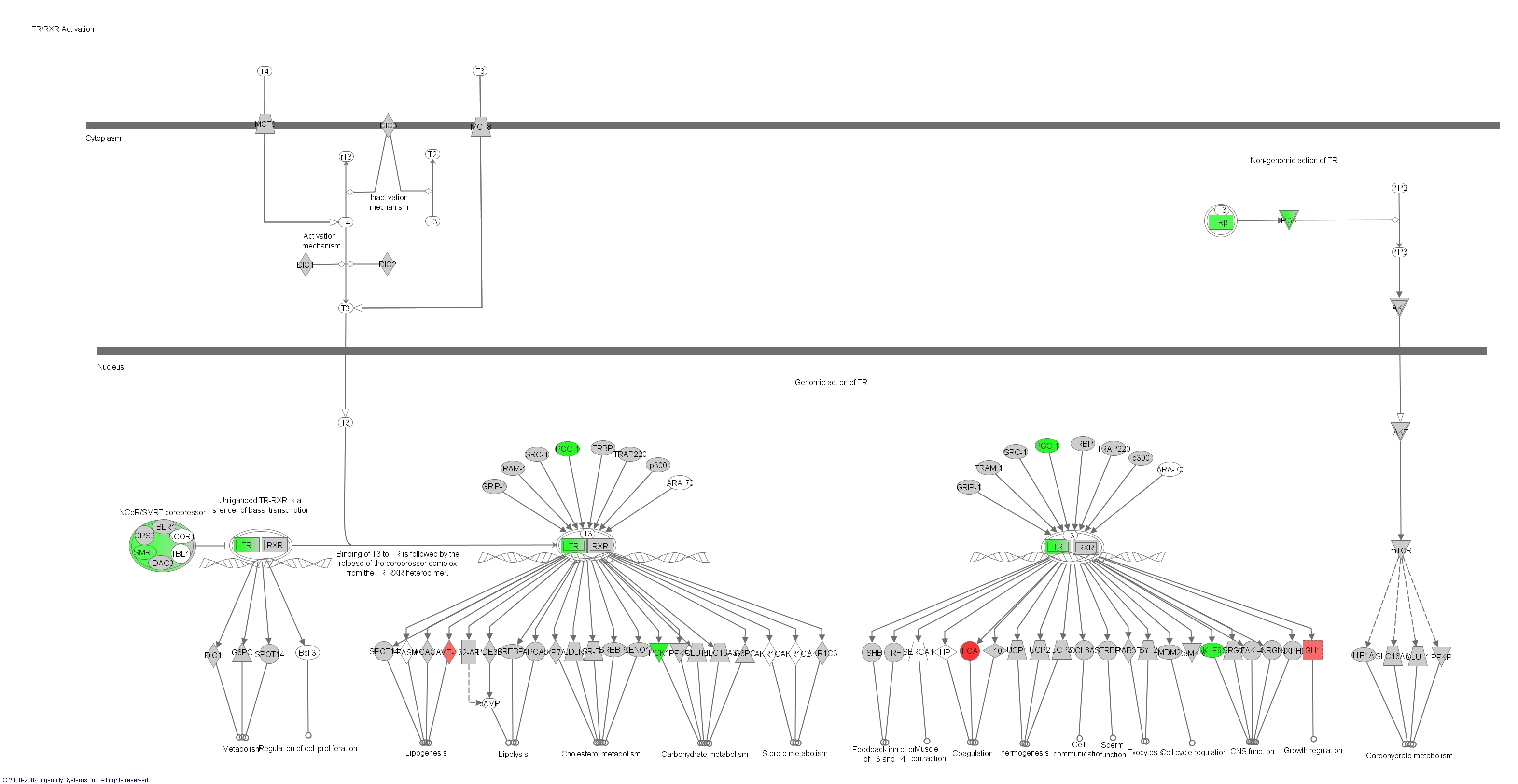

Supplement: Additional File 20 — Thyroid pathway in ileum (Ileum_thyroid_pathway.jpg). Images from IPA for the TR/RXR pathway for the ileum. Red shading indicates increased mRNA amounts of the respective gene during lactation, and green shading indicates decreased amounts of mRNA. [file 1471-2164-12-95-S20.JPEG]
