# Supplementary material for: Differential gene expression in liver and small intestine from lactating rats compared to age-matched virgin controls detects increased mRNA of cholesterol biosynthetic genes
Source: BMC Genomics. 2011 Feb 3;12:95. doi: 10.1186/1471-2164-12-95 (PMC3045338; doi:10.1186/1471-2164-12-95)
Supplement: Additional File 4 — Volcano plots (Volcano_plots.doc). Volcano plots comparing the log2 fold changes (reported as mean untransformed lactating intensity divided by untransformed mean control intensity) against the calculated pairwise comparison p-value for each individual tissue in .doc format. Volcano plots are for A) Liver, B) Duodenum, C) Jejunum, and D) Ileum. Each tissue responded differently to lactation. The blue line indicates the significance cutoff of p < 0.01. The number of differentially expressed genes were 420 in the liver, 337 in the duodenum, 402 in the jejunum, and 523 in the ileum, when an overall treatment main effect p-value cutoff of p < 0.05 was incorporated. Of particular note is a series of genes that were strongly downregulated in the duodenum (Additional File 1 pattern -100; discussed in Results.) [file 1471-2164-12-95-S4.DOC]

A

B

C

D
