# Supplementary material for: Differential gene expression in liver and small intestine from lactating rats compared to age-matched virgin controls detects increased mRNA of cholesterol biosynthetic genes
Source: BMC Genomics. 2011 Feb 3;12:95. doi: 10.1186/1471-2164-12-95 (PMC3045338; doi:10.1186/1471-2164-12-95)
Supplement: Additional File 6 — Genes with decreased mRNA in all tissues (Genes_with_decreased_mrna_all_tissues.doc). To be considered part of a grouping, genes must have a physiologic state p < 0.05 and at least one tissue simple effect p < 0.01. Reported p-values are tissue simple effect p-values and represent the comparison between Lactation and Control in the corresponding tissue. For the purposes of assigning patterns, the significance cutoff for the remaining tissue simple effect was set to p < 0.05. Abbreviations used as in Table 1. *Gene is at the Extended confidence level. [file 1471-2164-12-95-S6.DOC]

| Transcript Cluster ID | Gene Symbol | R L | R D | R J | R IL |
| --- | --- | --- | --- | --- | --- |
| 7040487 | Kruppel-like factor 9 | 0.74  (p=0.0014) | 0.59  (p=1.9e-06) | 0.58  (p-9.7e-07) | 0.48  (p=7.5e-09) |
| 7078982 | Chromodomain helicase DNA binding protein 3* | 0.76  (p=2.1e-05) | 0.71  (p=1.8e-06) | 0.80  (p=0.0004) | 0.77  (p=0.0001) |
| 7078988 | Jumonji domain containing 3 predicted* | 0.85  (p=0.0014) | 0.84  (p=0.0006) | 0.87  (p=0.0048) | 0.79  (p=1.5e-05) |
| 7092094 | Bromodomain and WD repeat containing 1_predicted* | 0.71  (p=0.0026) | 0.80  (p=0.0393) | 0.76  (p=0.0092) | 0.72  (p=0.0032) |
| 7092549 | RGD1562717_predicted* | 0.75  (p=0.0003) | 0.84  (p=0.0090) | 0.75  (p=0.0002) | 0.81  (p=0.0041) |
| 7094537 | Coiled-coil domain containing 50 | 0.75  (p=0.0007) | 0.84  (p=0.0212) | 0.68  (p=3.0e-05) | 0.77  (p=0.0017) |
| 7116362 | WD repeat and FYVE containing 3 predicted* | 0.81  (p=0.0086) | 0.85  (p=0.0340) | 0.86  (p=0.0465) | 0.78  (p=0.0022) |
| 7129810 | Calcium/calmodulin-dependent protein kinase II gamma | 0.76  (p=0.0047) | 0.83  (p=0.0350) | 0.61  (p=7.4e-06) | 0.57  (p=1.2e-06) |
| 7140113 | Leucine-rich repeats and calponin homology (CH) domain containing 1* | 0.87  (p=0.0252) | 0.84  (p=0.0072) | 0.86  (p=0.0147) | 0.81  (p=0.0013) |
| 7166075 | RGD1560155_predicted* | 0.82  (p=-.0060) | 0.83  (p=0.0105) | 0.86  (0.0343) | 0.78  (p=0.0011) |
| 7171399 | Mucosa associated lymphoid tissue lymphoma translocation gene 1_predicted* | 0.86  (p=0.0219) | 0.87  (p=0.0456) | 0.76  (p=0.0002) | 0.86  0.0214) |
| 7179785 | Nuclear factor I/X (CCAAT-binding transcription factor)* | 0.73  (p=4.8e-08) | 0.86  (p=0.0007) | 0.87  (p=0.0019) | 0.85  (p=0.0003) |
| 7198002 | Sortillin 1* | 0.85  (p=0.0119) | 0.87  (p=0.207) | 0.78  (p=0.0003) | 0.77  (p=0.0003) |
| 7219624 | REV3-like, catalytic subunit of DNA polymerase zeta (yeast)* | 0.80  (p=0.0346) | 0.79  (p=0.0174) | 0.69  (p=0.0009) | 0.74  (p=0.0045) |
| 7230017 | Low density lipoprotein receptor-related protein 4 | 0.85  (p=0.0250) | 0.87  (p=0.0405) | 0.79  (p=0.0019) | 0.77  (p=0.0010) |
| 7243436 | RAS guanyl releasing protein 1 (calcium and DAG-regulated) | 0.81  (p=0.0202) | 0.82  (p=0.0275) | 0.67  (p=0.0004) | 0.74  (p=0.0032) |
| 7245188 | Solute carrier family 23 (nucleobase transporters), member 2 | 0.74  (p=0.0005) | 0.70  (p=0.0001) | 0.74  (p=0.0002) | 0.72  (p=-.0006) |
| 7247292 | RGD1562582_predicted* | 0.81  (p=0.0277) | 0.81  (p=0.0265) | 0.73  (p=0.0014) | 0.80  (p=0.0135) |
| 7250393 | ATP-binding cassette, sub-family B (MDR/TAP), member 1 | 0.63  (p=0.0011) | 0.66  (p=0.0016) | 0.65  (p=0.0022) | 0.70  (p=0.0055) |
| 7260613 | Myeloid/lymphoid or mixed-lineage leukemia 5 (trithorax homolog, Drosophila)* | 0.77  (p=0.0093) | 0.80  (p=0.0192) | 0.77  (p=0.0064) | 0.76  (p=0.0076) |
| 7261911 | Pyruvate dehydrogenase kinase, isozyme 4 | 0.52  (p=0.0013) | 0.55  (p=0.0030) | 0.64  (p=0.0193) | 0.60  (p=0.0082) |
| 7278654 | Fatty acid amide hydrolase | 0.86  (p=0.0154) | 0.89  (p=0.0460) | 0.84  (p=0.0095) | 0.83  (p=0.0049) |
| 7297274 | Ataxin 7-like 1* | 0.71  (p=1.5e-06) | 0.89  (p=0.0341) | 0.89  (p=0.0383) | 0.81  (p=0.0007) |
| 7305576 | Phosphoinositide-3-kinase, catalytic, gamma polypeptide * | 0.82  (p=0.0462) | 0.77  (p=0.0048) | 0.69  (p=0.0003) | 0.67  (p=0.0002) |
| 7313779 | PCTAIRE protein kinase 2* | 0.79  (p=0.0087) | 0.80  (p=0.0108) | 0.82  (p=0.203) | 0.77  (p=0.0047) |
| 7318132 | Zinc finger protein 41 predicted* | 0.85  (p=0.0031) | 0.87  (p=0.0089) | 0.85  (p=0.0056) | 0.85  (p=0.0036) |
| 7322282 | Cold inducible RNA binding protein | 0.72  (p=0.0039) | 0.76  (p=0.0128) | 0.65  (p=0.0006) | 0.58  (p=0.0001) |
| 7326271 | Low density lipoprotein-related protein 1* | 0.69  (p=1.5e-05) | 0.62  (p=5.0e-07) | 0.65  (p=2.0e-06) | 0.65  (p=1.7e-06) |
| 7338021 | RGD1310552_predicted* | 0.69  (p=0.0001) | 0.84  (p=0.0295) | 0.74  (p=0.0010) | 0.65  (p=1.2e-05) |
| 7355138 | LOC301455* | 0.65  (p=0.0001) | 0.82  (p=0.0323) | 0.81  (p=0.0227) | 0.76  (p=0.0039) |
| 7376078 | O-linked N-acetylglucosamine (GlcNAc) transferase (UDP-N-acetylglucosamine:polypeptide-N-acetylglucosaminyl transferase) | 0.75  (p=0.0095) | 0.75  (p=0.0080) | 0.62  (p=4.9e-05) | 0.73  (p=0.0047) |
