# Supplementary material for: Differential gene expression in liver and small intestine from lactating rats compared to age-matched virgin controls detects increased mRNA of cholesterol biosynthetic genes
Source: BMC Genomics. 2011 Feb 3;12:95. doi: 10.1186/1471-2164-12-95 (PMC3045338; doi:10.1186/1471-2164-12-95)
Supplement: Additonal File 11 — Genes regulated by Srebp proteins (Genes_Regulated_by_Srebp_proteins.doc). Genes that increase expression in Srebp-1a overexpressing mice and Srebp-2 overexpressing mice, and decrease expression in Scap knockout mice [34]. Overrepresentation analysis showed that genes in this list occurred more frequently than expected by chance in the lists of differentially expressed genes (p < 1 × 10^-4^ in each tissue.) Abbreviations used as in Table 1. *Gene is at the Extended confidence level. [file 1471-2164-12-95-S11.DOC]

| **Gene Symbol** | **R L** | **R D** | **R J** | **R IL** | **p<0.01** |
| --- | --- | --- | --- | --- | --- |
| Acetoacetyl Coenzyme A synthetase | 1.92  (p=3.13e-05) | 1.45  (p=0.0077) | 1.03  (p=0.87) | 1.29  (p=0.075) | L,D |
| Acyl-CoA synthetase short-chain family member 2 | Not at extended level. |  |  |  | - |
| ATP citrate lyase | 1.23  (p=0.030) | 1.27  (p-0.026) | 1.01  (p=0.87) | 1.17  (p=0.13) | - |
| 7-dehydrocholesterol reductase | 1.54  (p=0.00060) | 1.54  (p=0.00066) | 0.89  (p=0.30) | 1.41  (p=0.0030) | L,D,IL |
| Farnesyl diphosphate synthase | Not at extended level |  |  |  | - |
| 3-hydroxy-3-methylglutaryl-Coenzyme A synthase 1 | 1.48  (p=0.00049) | 1.60  (p=7.38e-05) | 1.13  (p=0.17) | 1.55  (p=0.00021) | L,D,IL |
| 3-hydroxy-3-methylglutaryl-Coenzyme A reductase | 1.97  (p=6.15e-06) | 1.64  (p=0.00018) | 1.17  (p=0.13) | 1.38  (p=0.0072) | L,D,IL |
| Hydroxysteroid (17-beta) dehydrogenase 7 | 1.70  (p=2.1E-06) | 1.53  (p=4.73E-05) | 1.19  (p=0.045) | 1.50  (p=9.11E-05) | L,D,IL |
| Isopentenyl-diphosphate delta isomerase 1 | 1.60  (p=0.00061) | 2.11  (p=2.84E-06) | 1.33  (p=0.023) | 1.91  (p=2.58E-05) | L,D,IL |
| Cytochrome p450 51 | 1.24  (p=0.012) | 2.14  (p=1.79E-09) | 1.50  (p=4.22E-05) | 1.89  (p=6.8E-08) | D,J,IL |
| Low density lipoprotein receptor | 1.03  (p=0.83) | 1.63  (p=0.0042) | 1.87  (p=0.00076) | 1.29  (0.080) | D,J |
| Mevalonate (diphospho) decarboxylase | 2.65  (p=3.84E-05) | 2.15  (p=0.00041) | 1.49  (p=0.032) | 1.73  (p=0.0042) | L,D,IL |
| NAD(P) dependent steroid dehydrogenase-like | 1.08  (p=0.16) | 1.70  (p=3.95E-09) | 1.14  (p=0.024) | 1.52  (p=2.57E-07) | D,IL |
| Phosphomevalonate kinase | 1.91  (p=0.031) | 1.34  (p=0.16) | 1.15  (p=0.68) | 1.48  (p=0.17) | - |
| Lanosterol synthase | 2.07  (p=0.0010) | 1.65  (p=0.0091) | 1.32  (p=0.082) | 1.47  (p=0.026) | L,D |
| Squalene Epoxidase | 1.83  (p=2.35E-05) | 2.27  (p=5.58E-07) | 1.72  (p=0.00013) | 1.70  (p=0.00025) | L,D,J,IL |
| Farnesyl diphosphate farnesyl transferase 1 | 1.38  (p=0.00091) | 1.98  (p=4.65E-08) | 1.42  (p=0.00041) | 1.55  (p=4.13E-05) | L,D,J,IL |
| Sterol-C4-methyl oxidase-like | 1.41  (p=0.0015) | 2.19  (p=1.94E-07) | 1.45  (p=0.00082) | 1.80  (p=8.23E-06) | L,D,J,IL |
| Sterol c5 desaturase | 1.12  (p=0.13) | 1.44  (p=5.5E-05) | 1.10  (p=0.20) | 1.41  (p=0.00014) | D,IL |
| Transmembrane 7superfamily member 2 | 1.08  (p=0.47) | 1.24  (p=0.076) | 1.16  (p=0.26) | 1.33  (p=0.017) | - |
| Insulin induced gene 1 | 1.14  (p=0.14) | 2.40  (p=2.03E-07) | 1.59  (p=0.00079) | 1.45  (p=0.0044) | D,J,IL |
| proprotein convertase subtilisin/kexin type 9 | 1.16  (p=0.38) | 1.44  (p=0.03) | 1.28  (p=0.12) | 1.97  (p=0.00044) | IL |
| StAR-related lipid transfer (START) domain containing 4 | Not at extended level |  |  |  | - |
| Acetyl-Coenzyme A carboxylase alpha | 1.53  (p=0.0010) | 1.26  (p=0.059) | 0.83  (p=0.13) | 1.20  (p=0.16) | L |
| Cytochrome b5 type b | 1.15  (p=0.09) | 1.02  (p=0.81) | 0.96  (p=0.59) | 1.28  (p=0.0047) | IL |
| Fatty acid synthase | Not at extended level |  |  |  | - |
| Elongation of very long chain fatty acids)family member 6 (yeast) | 3.27  (p=1.96E-06) | 1.13  (p=0.46) | 1.41  (p=0.060) | 1.54  (p=0.19) | L |
| (Malic enzyme 1 NADP+ dependent) | 1.39  (p=0.00093) | 1.02  (p=0.82) | 1.02  (p=0.834) | 1.32  (p=0.0058) | L,IL |
| Sterol regulatory element binding factor 1* | 1.05  (p=0.67) | 1.21  (p=0.13) | 1.64  (p=0.00066) | 1.09  (p=0.46) | J |
| Aldolase C, fructose-bisphosphate | 1.73  (p=1.53E-07) | 1.18  (p=0.47) | 1.16  (p=0.067) | 0.96  (p=0.63) | L |
| Transmembrane protein 97 | 1.92  (p=6.92E-05) | 2.03  (p=2.79E-05) | 1.82  (p=0.00021) | 2.15  (p=8.66E-06) | L, D,J,IL |
| Retinol dehydrogenase 11 (all-trans/9-cis/11cis) | 1.33  (p=0.00029) | 1.25  (p=0.0031) | 1.01  (p=0.85) | 1.21  (p=0.011) | L,D |
| Mid1 interacting protein 1 (gastrulation specific G12 homolog (zebrafish)) | 1.25  (p=0.026 | 1.19  (p=0.10) | 0.93  (p=0.41) | 1.23  (p=0.046) | - |
