# Supplementary material for: Differential gene expression in liver and small intestine from lactating rats compared to age-matched virgin controls detects increased mRNA of cholesterol biosynthetic genes
Source: BMC Genomics. 2011 Feb 3;12:95. doi: 10.1186/1471-2164-12-95 (PMC3045338; doi:10.1186/1471-2164-12-95)
Supplement: Additional File 12 — Members of the Slc superfamily (Slcs.doc). Table displaying members of the Slc superfamily. The p < 0.01 column indicates in which tissues a change was detected. Abbreviations are as defined for Table 1. * Gene is at the Extended level of confidence. aSubstrates taken from the SLC tables database (http://www.bioparadigms.org/) [file 1471-2164-12-95-S12.DOC]

| **Gene Symbol** | **Probeset ID** | **R L** | **R D** | **R J** | **R IL** | **p<0.01** | **Substrate^a^** |
| --- | --- | --- | --- | --- | --- | --- | --- |
| Slc1a2 | 7230622 | 1.855  (p=4.00E-07) | 1.09  (p=0.338) | 1.08  (p=0.444) | 1.23  (p=0.034) | L | L-Glu, D/L-Asp |
| Slc2a8 | 7237701 | 1.046  (p=0.645) | 1.27  (p=0.01) | 1.26  (p=0.011) | 1.18  (p=0.074) | D | Glucose,  Fructose,  Galactose |
| Slc4a1 | 7083011 | 1.009  (p=0.887) | 1.4  (p=3.00E-04) | 1.16  (p=0.072) | 1.16  (p=0.061) | D | Chloride Bicarbonate |
| Slc4a9 | 7169367 | 1.106  (p=0.029) | 1.08  (p=0.122) | 1.14  (p=0.009) | 0.97  (p=0.566) | J | Inconclusive |
| Slc4a10 | 7227986 | 1.048  (p=0.733) | 0.96  (p=0.665) | 0.65  (p=0.002) | 0.88  (p=0.318) | J | Inconclusive |
| Slc5a5 | 7144113 | 1.14  (p=0.122) | 1  (p=0.969) | 1.65  (p=4.00E-06) | 1.28  (p=0.012) | J | I^-^ (ClO_4_^-^, SCN^-^,  NO_3_^-^, Br^-^) |
| Slc6a6 | 7256210 | 0.612  (p=7.00E-05) | 0.91  (0.337) | 0.93  (0.386) | 0.92  (0.368) | L | taurine |
| Slc6a9 | 7278898 | 0.775  (0.001) | 0.93  (273) | 0.91  (0.202) | 0.88  (0.067) | L | glycine |
| Slc6a20 | 7351229 | 0.856  (p=0.181) | 0.53  (p=4.00E-06) | 0.48  (p=5.00E-07) | 0.74  (p=0.008) | D,J,IL | imino acids (proline, pipecolate, sarcosine) |
| Slc7a9 | 7030676 | 1.242  (p=0.002) | 1.21  (p=0.005) | 1.04  (p=0.49) | 1.16  (p=0.027) | L, D | cationic amino acids, large neutral amino acids (system b^0,+^-like) |
| Slc7a15* | 7304464 | 0.72  (p=0.125) | 1.66  (p=0.018) | 0.33  (p=2.00E-05) | 0.46  (p=5.00E-04) | J, IL |  |
| Slc11a2 | 7331795 | 1.017  (p=0.815) | 1.63  (p=2.00E-06) | 2.15  (p=2.00E-09) | 0.79  (p=0.008) | D,J,IL | Fe^2+^, Cd^2+^, Co^2+^, Cu^1+^, Mn^2+^ |
| Slc12a6* | 7231152 | 0.865  (p=0.145) | 0.86  (p=0.111) | 0.82  (p=0.042) | 0.72  (p=0.002) | Il | Potassium,  Chloride |
| Slc13a1 | 7262846 | 0.995  (p=0.992) | 0.82  (p=0.046) | 0.51  (p=3.00E-07) | 1.16  (p=0.046) | J | Sulfate, selenate, thiosulfate |
| Slc13a4 | 7263646 | 0.469  (p=3.00E-09) | 0.93  (p=0.373) | 1.09  (p=0.305) | 0.97  (p=0.759) | L | Sulfate |
| Slc16a7 | 7326050 | 0.724  (p=0.015) | 0.66  (p=0.003) | 0.76  (p=0.037) | 1.12  (p=0.364) | D | Pyruvate, lactate, ketone bodies |
| Slc16a13 | 7079308 | 1.198  (p=0.116) | 1.27  (p=0.058) | 1.54  (p=0.002) | 1.02  (p=0.75) | J |  |
| Slc17a2_predicted* | 7157791 | 0.668  (p=7.00E-04) | 0.73  (p=0.007) | 0.67  (p=0.001) | 0.96  (p=0.746) | L,D,J | unknown |
| Slc22a1 | 7027120 | 0.73  (p=0.024) | 0.85  (p=0.194) | 0.62  (p=0.002) | 0.74  (p=0.023) | J | Organic cations, polyspecific |
| Slc23a2 | 7245188 | 0.737  (p-5.00E-04) | 0.7  (p=8.00E-05) | 0.72  (p=2.00E-04) | 0.74  (p=6.00E-04) | L,D,J,Il | L-Ascorbic acid |
| Slc25a23* | 7367943 | 0.71  (p=0.026) | 0.81  (p=0.122) | 0.67  (p=0.009) | 0.67  (p=0.009) | J, IL | ATP, ADP, AMP, and Pi |
| Slc25a25 | 7237652 | 0.453  (p=5.00E-05) | 0.89  (p=0.436) | 0.89  (p=0.5) | 0.88  (p=0.352) | L |  |
| Slc26a3 | 7297162 | 0.969  (p=0.636) | 0.87  (p=0.039) | 0.75  (p=2.00E-04) | 0.82  (p=0.005) | J, IL | SO_4_^2-^, Cl^-^, HCO_3_^-^, OH^-^, oxalate |
| Slc26a8_predicted* | 7221187 | 0.661  (p=0.001) | 1  (p=0.863) | 0.69  (p=.003) | 0.78  (p=0.033) | L,J | SO_4_^2-^, Cl^-^, oxalate |
| Slc28a2 | 7244077 | 1.04  (p=0.736) | 0.92  (p=0.311) | 1.59  (p=1.00E-05) | 1.15  (p=0.115) | J | Purine nucleosides, uridine |
| Slc30a3 | 7295580 | 1.737  (p=1.00E-05) | 1.07  (p=0.507) | 1.24  (p=0.032) | 0.85  (p=0.126) | L | Zinc |
| Slc30a6_predicted* | 7303630 | 1.037  (p=0.676) | 1.3  (p=0.004) | 1.11  (p=0.189) | 1.12  (p=0.201) | D | Zinc (by association only) |
| Slc34a2 | 7126150 | 0.651  (p=0.037) | 13.7  (p=1.00E-10) | 3.05  (p=3.00E-05) | 1.82  (p=0.023) | D,J | inorganic  phosphate |
| Slc35b1 | 7071470 | 1.239  (p=0.002) | 1.11  (p=0.094) | 1.01  (p=0.846) | 1.11  (p=0.093) | L |  |
| Slc38a7 | 7178572 | 1.137  (p=0.159) | 1.2  (p=039) | 1.3  (p=0.06) | 1.03  (p=0.692) | J |  |
| Slc39a4_predicted* | 7329323 | 2.814  (p=2.00E-10) | 1.89  (p=1.00E-07) | 1.65  (p=2.00E-06) | 1.68  (p=1.00E-06) | L,D,J,IL | Zinc |
| Slc39a5_predicted* | 7321347 | 0.625  (p=9.00E-04) | 0.94  (p=0.549) | 0.88  (p=0.206) | 0.79  (p=0.048) | L |  |
| Slc40a1 | 7361893 | 0.79  (p=0.105) | 1.17  (p=0.263) | 1.97  (p=7.00E-05) | 1.45  (p=0.012) | J | Ferrous iron |
| Slco1a6 | 7270905 | 0.984  (p=0.902) | 1.22  (p=0.047) | 1.53  (p=2.00E-04) | 1.1  (p=0.311) | J |  |
| Slco2b1 | 7054899 | 0.852  (p=0.205) | 0.81  (p=0.119) | 0.66  (p=0.008) | 0.71  (p=0.022) | J | E-3-S, DHEAS, BSP |
