# Supplementary material for: Differential gene expression in liver and small intestine from lactating rats compared to age-matched virgin controls detects increased mRNA of cholesterol biosynthetic genes
Source: BMC Genomics. 2011 Feb 3;12:95. doi: 10.1186/1471-2164-12-95 (PMC3045338; doi:10.1186/1471-2164-12-95)
Supplement: Additional File 21 — RT-PCR primers (RT_PCR_primers.doc). Primer sequences for all genes analyzed by RT-PCR. [file 1471-2164-12-95-S21.DOC]

Sqle Probe #12

SqleF

Ctgtcagaatgctcgtctgc

SqleR

Cgcatctcccagaagtagga

Ugt2b4 Probe #69

UGT2b4-1285F

tcaaagtcagatatgctcaatgc

Ugt2b4-1362R

tgacaaccacatagcattcttttta

Tmem97 probe #92

Tmem124F:

Cgaactctacccgcagga

Tmem224R:

Ggaaggacttgaaccacacag

Npc1l1 Probe #56

Npc1l1-257F

ccacttttgcctgttgctct

Npc1l1-316R

Tgatggacatgctgctttct

Fdft1 Probe #65

RFdft1F

gcaaggagaagcaccgagta

RFdft1R

ttctaaactccagggagatcgt

Slc39a4 Probe#25

RSlc39a4F

aacccaccagggaggaga

RSlc39a4R

ttctggaaacccctgcttc

Cyp1a1 probe#76

RCyp1a1F

tggggtcctagagaacactctt

RCyp1a1R

cacagaaggcatgatctaggtg

CYP3a23/3a1 probe #113

RCyp3a23/3a1F

Gaaactgcaggaggagatcg

RCyp3a23/3a1R

tcacagtatcataggtgggaggt

Abcb1a probe#78

Abcb1aF

gcaaatgtaggaaacaaccgtag

Abcb1aR

gtaggcgtacgtggtcatttc

Hmgcr probe#1

HmgcrF

cagtgatggagccacatgaa

HmgcrR

Aactcctggccacaggaac

Ctsbf (upl#62)

Aatgtggaggtgtctgctgag

 CtsbR

Gggatagccaccattacagc

Tmbim6F (UPL#125)

 Cgaagaggtgggctaagaag

 Tmbim6R

Gggaaaattttaagagggcatc

Tmed2F (UPL#21 )

Ccgtaaagcacgaacaggag

 Tmed2R

ttcgaagaaggaccaaagga
